# Evaluation of the neurotrophic peptide mixture in pathogenetic therapy of patients with Parkinson’s disease

**DOI:** 10.1038/s41531-026-01270-6

**Published:** 2026-01-23

**Authors:** Dmytro Krasnienkov, Iryna Karaban, Nina Karasevych, Nataliia Melnyk, Sergiy Kryzhanovskyi, Kateryna Rozova, Olga Gonchar, Iryna Mankovska, Sofiia Smovzh, Kostiantyn Midlovets, Olexiy Barsukov, Oksana Zabuha, Tetiana Papurina

**Affiliations:** 1 D.F. Chebotarev Institute of Gerontology of the National Academy of Medical Sciences of Ukraine, Kyiv, Ukraine; 2 Blackthorn AI Ltd, London, UK; 3 Bogomoletz Institute of Physiology of the National Academy of Sciences of Ukraine, Kyiv, Ukraine; 4 Preci LLC, Bila Tserkva, Ukraine; 5 V. N. Karazin Kharkiv National University, Kharkiv, Ukraine; 6 M.H. Kholodny Institute of Botany, Kyiv, Ukraine

**Keywords:** Computational biology and bioinformatics, Genetics, Medical research, Neurology, Pathogenesis

## Abstract

This exploratory, single-group, open-label study investigated 17 patients with Parkinson’s disease (PD) using a pre-post design. Motor and non-motor outcomes were assessed through clinical scales, biochemical and genetic analyses, and machine learning models (Gradient Boosting Machines, Random Forests). After treatment with a neurotrophic peptide mixture, improvements were observed in daily activity (16%), cognition (11%), depression (10% reduction), and reactive anxiety (23% reduction). Biological changes included a 45% increase in platelet δ-granules, higher mitochondrial counts, elevated gene expression (notably BDNF in women, p = 0.046), and modulation of oxidative stress markers (17% reduction in TBARS, 30% increase in GSH). Machine learning identified BDNF and PINK1 expression, along with MOCA and MMSE scores, as key predictors of UPDRS improvement. These findings suggest that neurotrophic peptide therapy may influence clinical, structural, and molecular domains in PD. Larger, controlled trials are warranted to confirm therapeutic potential and clarify associations with cognitive and neurotrophic parameters.

## Introduction

Parkinson’s disease is a neurodegenerative condition typically observed in older individuals, characterized by a gradual onset and progressive nature. The initial manifestation often includes tremors, followed by symptoms such as slowed movement (bradykinesia) and muscle stiffness (rigidity)^1^. The disease is linked to the degeneration of dopaminergic neurons in the substantia nigra, the presence of alpha-synuclein aggregates within neuronal cells (Lewy bodies and Lewy neurites), and subsequent pathology in regions like the basal forebrain, amygdala, and medial temporal lobe structures, progressing to cortical areas in advanced stages^2^.

Mitochondrial abnormalities and dysfunction are recognized as key contributors to Parkinson’s disease pathogenesis in a subset of patients^3^. Some data was obtained on the availability correlation of the functional activity of mitochondria in lymphocytes, which displays multisystem mitochondrial failure and indicates of energy dysfunction in general^4^. The spectrum of mitochondrial dysfunctions in PD includes dysfunction of the respiratory chain (inhibition of the NADH-oxidase oxidation pathway), hyperproduction of ATP, disruption of Ca-regulatory systems, opening of the mitochondrial pore, perturbations of mitochondrial dynamics, and dysregulation of mitochondrial clearance^5^.

Although oxidative stress plays a significant role in the pathogenesis of PD, therapy based on the use of exogenous antioxidants has not been shown to be highly effective. An alternative strategy that can counteract the damaging effects of free radicals and restore cellular redox balance is the activation of endogenous antioxidant enzymes present in the central neural system, such as superoxide dismutase, catalase, and glutathione peroxidase. The transcription of these cytoprotective proteins is regulated by the transcription factor Nrf2, which plays a leading role in the regulation of cellular redox status and is the main regulator of the cellular response to oxidative stress through the induction of antioxidant and detoxifying enzymes and proteins^6^. This is consistent with our previous data, which showed that lipid peroxidation was significantly increased while reduced glutathione (GSH) and glutathione-dependent antioxidant enzymes activity markedly decreased in blood of patients with PD as well as in rat brain under modeling of PD^7^. Various activators of the Nrf2/ARE signaling pathway, including peptides, have shown their effectiveness in both in vitro and in vivo models of neurological disorders^8^.

The Neurotrophic peptide mixture is peptidergic mixture with neurotrophic-like properties, made from a mixture of low molecular weight, porcine-derived peptides and free amino acids. The pharmaceutical preparation includes the peptide fragments: nerve growth factor (NGF), brain-derived neurotrophic factor (BDNF), ciliary neurotrophic factor (CNTF), enkephalins, orexin, and P21^9^.

The use of the Neurotrophic peptide mixture significantly reduces neurological deficits, improves cognitive abilities, and reduces the volume of the infarction zone during stroke^10,11^. The Neurotrophic peptide mixture has an organ-specific multimodal effect on the brain, i.e., it provides metabolic regulation, neuroprotection, functional neuromodulation, and neurotrophic activity^12^. The Neurotrophic peptide mixture protects neurons from the damaging effects of lactic acidosis, prevents the formation of free radicals^13^, increases survival and prevents neuronal death under conditions of hypoxia and ischemia, and reduces the damaging neurotoxic effect of glutamate^14^.

Over the past decade, compelling evidence has emerged neuroprotective properties of the Neurotrophic peptide mixture, explaining its multi-level impact on both the pathological cascade of damage and on subsequent recovery processes^15^. The ability of the Neurotrophic peptide mixture to influence the processes of neuroprotection and neuroplasticity was proven in a series of studies^15–17^ on a model of Alzheimer’s disease in transgenic mice for the amyloid precursor protein (mThy-1-hAPP751 tg). Using a model of Alzheimer’s disease, it was shown that the Neurotrophic peptide mixture, through the regulation of the synthesis and transport of amyloid precursor protein and the deposition of β-amyloid in synapses, has a synaptotrophic effect, which plays a primary role in the processes of neuroplasticity, which are extremely important in the acquisition of new skills and the maintenance of existing ones^18^.

Increasing studies have demonstrated that the Neurotrophic peptide mixture administration can promote recovery of motor function, improve early brain injury, and decrease hippocampal neuronal death^19^. In recent clinical studies, the Neurotrophic peptide mixture improved overall outcomes after moderate to severe traumatic brain injury and was also safe and better for early rehabilitation patients after ischemic stroke^20^. Lu et al. reported that the Neurotrophic peptide mixture can improve neurological dysfunction and brain damage, relieve neuroinflammation and prevent apoptosis after brain injury^21^.

Based on the multifaceted mechanism of action of the Neurotrophic peptide mixture, including its effect on the processes of neurogenesis, neuroplasticity and neuroprotection, the drug currently has three main indications for use - stroke, dementia and traumatic brain injury^10,22,23^.

Luchanina et al. demonstrated for the first time the effect of Neurotrophic peptide mixture on motor activity and muscle tone in patients with PD treated with levodopa^13^. It was established that the Neurotrophic peptide mixture improved the engraftment of a DOPA transplant from stem cells in the substantia nigra^24^ and reduced the severity of symptoms in model animals^12^.

The current exploratory study dataset comprised clinical, cognitive, biochemical, and demographic information from PD patients undergoing the Neurotrophic peptide mixture therapy. Key features included cognitive assessments (MMSE, MOCA), neurotrophic markers (BDNF, PINK1), oxidative stress indicators (SOD, DJ1), and demographic factors (age, sex). The primary outcome was the change in UPDRS scores, serving as an objective measure of therapeutic response to the Neurotrophic peptide mixture. Also, in this study, machine learning models were employed to predict UPDRS score changes as a key indicator of the Neurotrophic peptide mixture’s therapeutic effectiveness, offering a data-driven evaluation of treatment response.

## Results

### Clinical and neurophysiological assessment of the effectiveness of course treatment

**Fig. 1:**
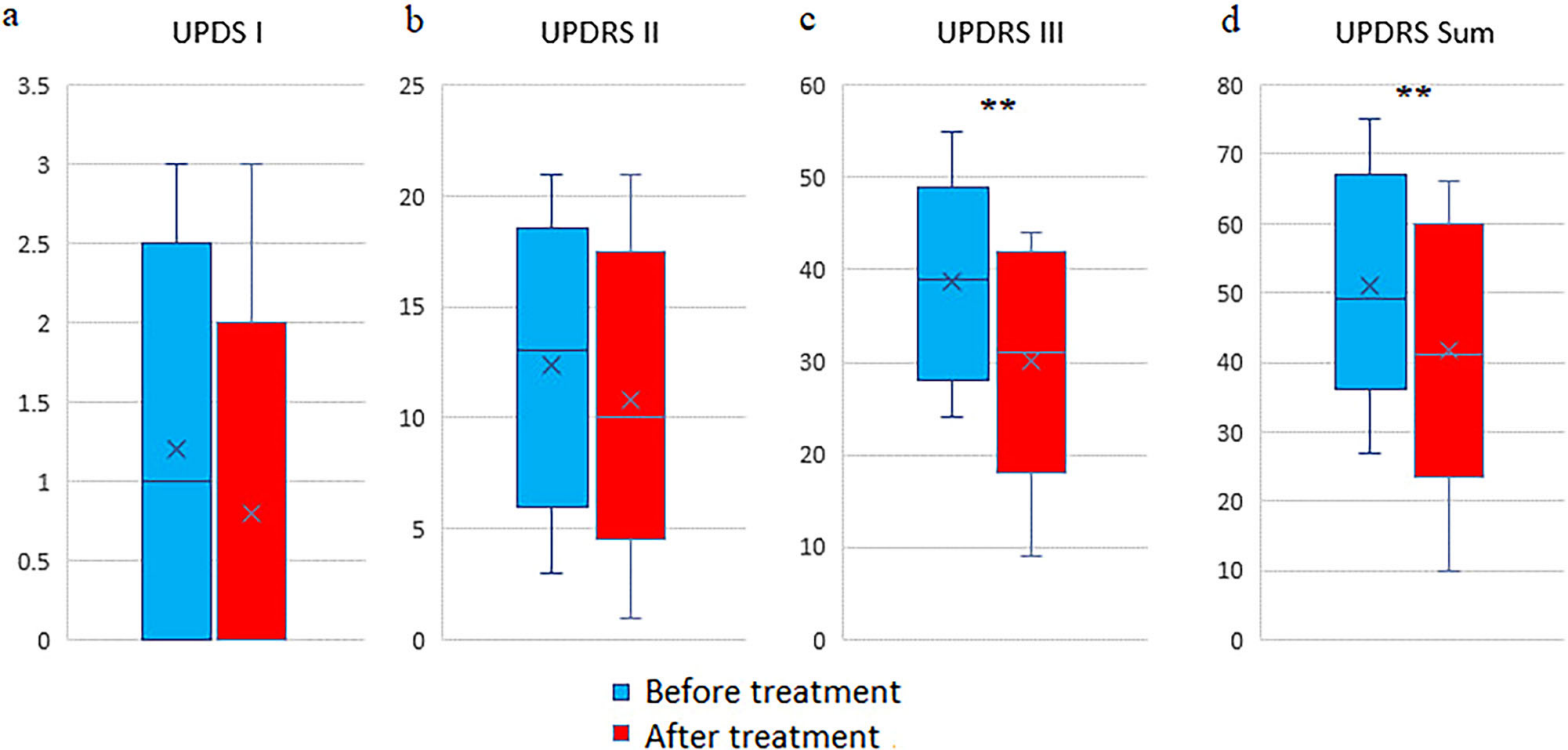
Effect of treatment with neurotrophic peptide mixture on Unified Parkinson’s Disease Rating Scale (UPDRS) scores. **a** UPDRS part I (mentation, behavior, mood) scores before and after treatment. **b** UPDRS part II (activities of daily living) scores. **c** UPDRS part III (motor examination) scores. Significant reduction was observed after treatment (*p* < 0.01). **d** Total UPDRS score (sum of parts I–III), also showing a significant decrease after treatment (*p* < 0.01). Blue bars represent values before treatment, red bars represent values after treatment. Data are shown as box plots with median (horizontal line), interquartile range (box), minimum and maximum values (whiskers), and mean (cross). Secondary/Exploratory outcomes: Descriptive statistics and effect sizes reported without inferential p-values.

A course of the Neurotrophic peptide mixture administration against the background of basic antiparkinsonian therapy had a beneficial effect on the condition of the majority of patients, primarily on their activity in everyday life and the ability to self-care. Objectively, this effect was expressed in a significant decrease in scores of all UPDRS subscales (~16% on average) (Fig. 1).

Both clinically central endpoints improved significantly (*p* < 0.01). Secondary measures (UPDRS I and UPDRS II) generally trend in the same direction; effect sizes are provided without formal inference.

**Fig. 2:**
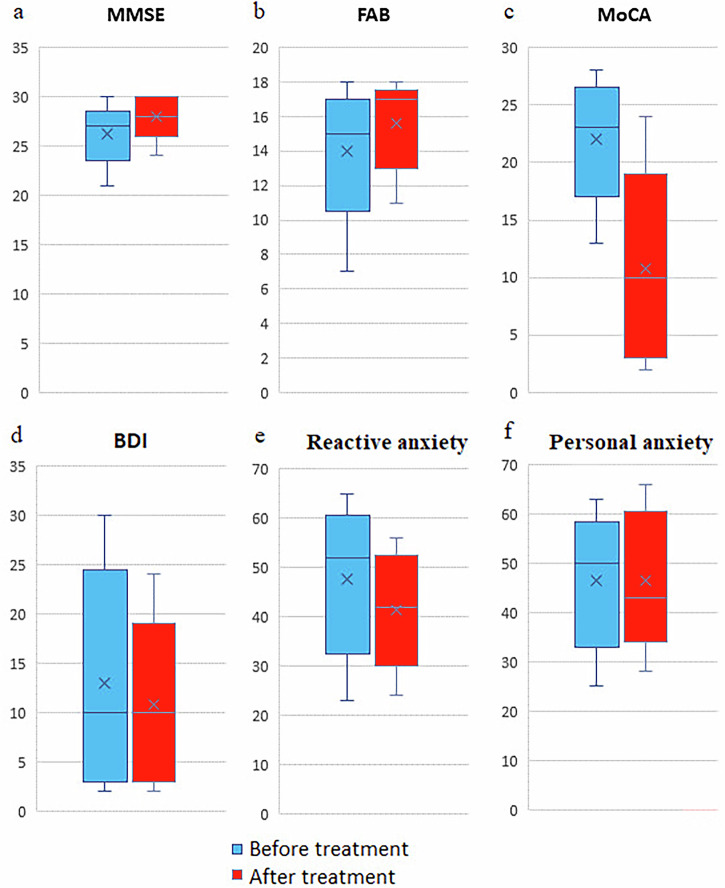
The effect of the Neurotrophic peptide mixture treatment on the cognitive functions, the emotional and motivational sphere. **a** MMSE (mental state) scores before and after treatment. **b** FAB (frontal dysfunction) scores before and after treatment. **c** MoCA (cognitive assessment) scores before and after treatment. **d** BDI (depression) scores before and after treatment. **e** Reactive anxiety (Spielberger-Hanin Scale) scores before and after treatment. **f** Personal anxiety (Spielberger-Hanin Scale) scores before and after treatment. Blue bars represent values before treatment, red bars represent values after treatment. Data are shown as box plots with median (horizontal line), interquartile range (box), minimum and maximum values (whiskers), and mean (cross). Secondary/Exploratory outcomes: Descriptive statistics and effect sizes reported without inferential p-values.

A beneficial impact was observed following administration of a regimen of the Neurotrophic peptide mixture on a spectrum of non-motor dysfunctions concomitant with the motor manifestations of Parkinson’s disease (PD). Participants exhibited statistically significant enhancements in cognitive abilities as assessed by specialized instruments including MMSE, FAB, and MoCA (~11% on average). Moreover, there was a notable reduction in scores indicative of depression measured via the Beck Depression Inventory scale (~10% on average), as well as in reactive anxiety assessed by the Spielberger-Hanin scale (~23% on average). Notably, levels of personal anxiety remained consistent relative to baseline measurements. As a result, all observed Secondary outcomes are trended in the direction of improvement (Fig. 2).

**Fig. 3:**
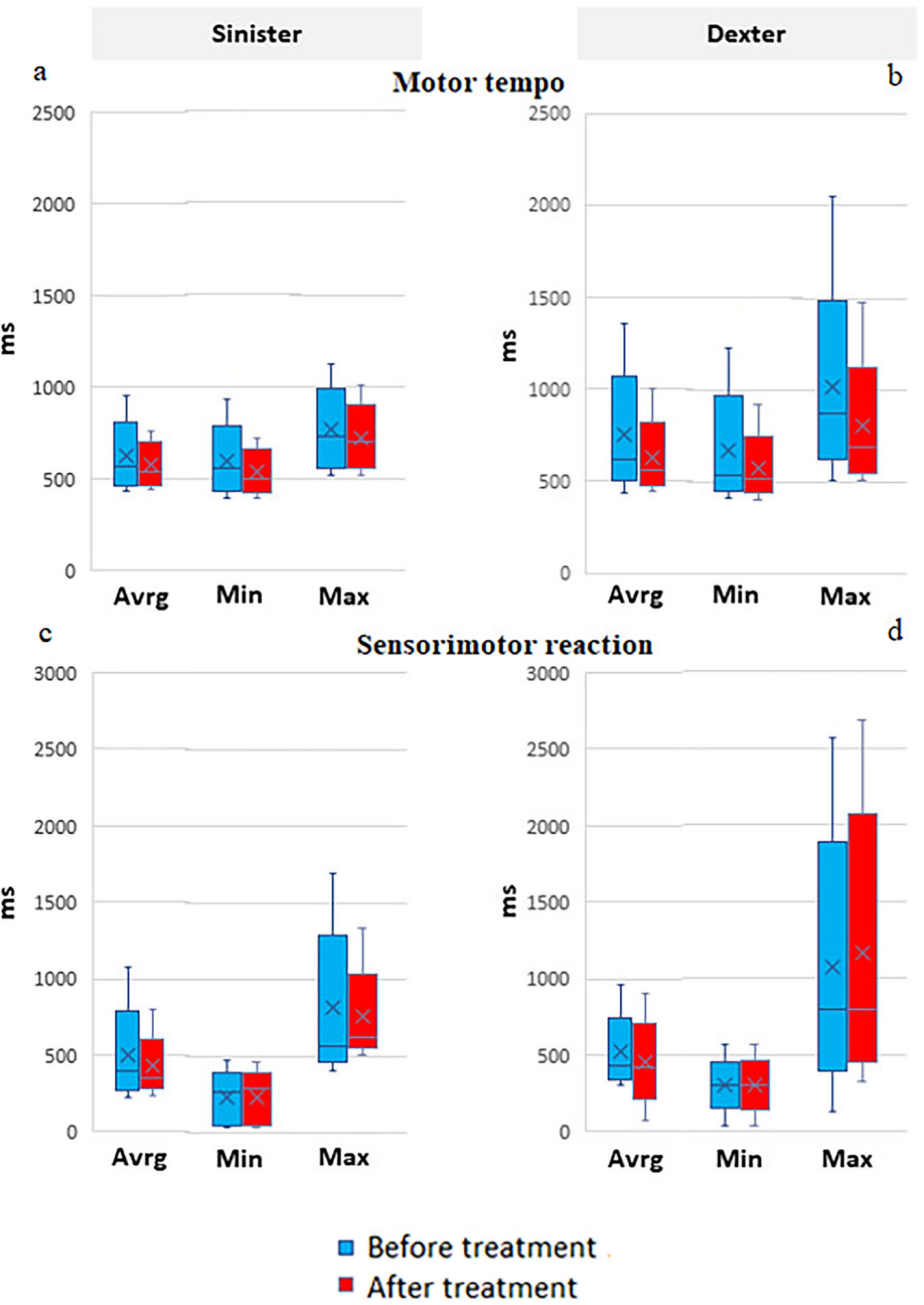
The effect of the Neurotrophic peptide mixture on the characteristics of motor activity in the Motor tempo and Sensorimotor reaction tests. **a**, **b** represent Motor tempo scores for Sinister and Dexter respectively. **c**, **d** represent Sensimotor reaction scores for Sinister and Dexter respectively. Blue bars represent values before treatment, red bars represent values after treatment. Data are shown as box plots with median (horizontal line), interquartile range (box), minimum and maximum values (whiskers), and mean (cross). Average (Avrg), minimum (Min) and Maximum (Max) values are represented on every box plot. Secondary/Exploratory outcomes: Descriptive statistics and effect sizes reported without inferential p-values.

For instrumental assessment of the Neurotrophic peptide mixture effect on motor functions, the Motor tempo test showed higher sensitivity. As a result, the significant decrease in the average, minimum and maximum time of motor reactions was recorded (Fig. 3).

Those changes are more characteristic of the reactions of the right hand, while for the left hand only the average value of the motor tempo decreased. It can be assumed that this asymmetry of effects is associated with the sidedness of the pathological process, since a significant part of patients (76% of the study group) demonstrated predominantly right-sided symptoms of PD. These findings show a valuable positive impact of Neurotrophic peptide mixture on neurophysiological parameters.

At the same time, the latent period of a simple sensorimotor reaction did not change during a course of the Neurotrophic peptide mixture administration (Fig. 3).

### Changes of the H-reflex and M-response after administration of a regimen of Сerebrolysin

**Fig. 4:**
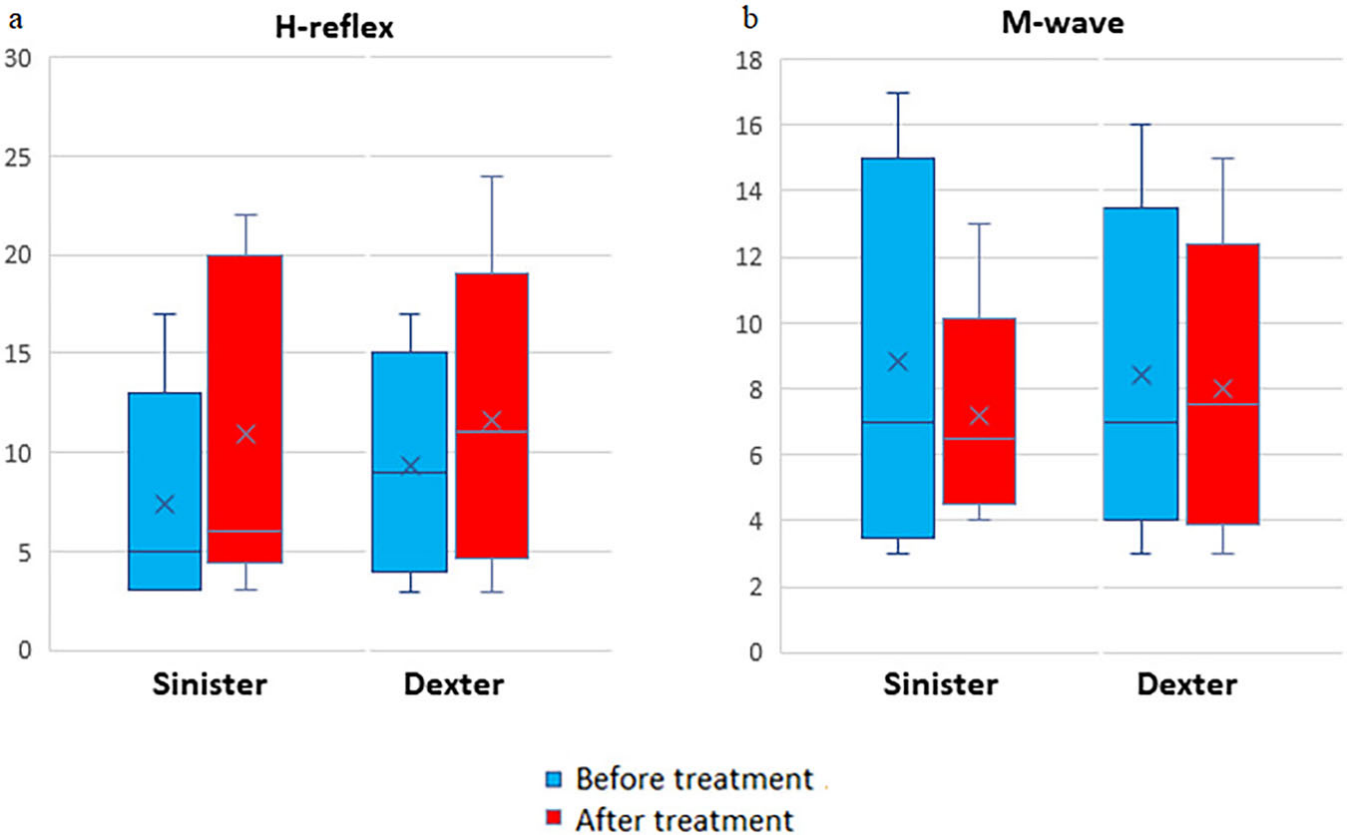
H-reflex and M-response thresholds before and after the course of the Neurotrophic peptide mixture. **a** H-reflex threshold scores for Sinister and Dexter before and after treatment. **b** M-response threshold scores for Sinister and Dexter before and after treatment. Blue bars represent values before treatment, red bars represent values after treatment. Data are shown as box plots with median (horizontal line), interquartile range (box), minimum and maximum values (whiskers), and mean (cross). Secondary/Exploratory outcomes: Descriptive statistics and effect sizes reported without inferential *p*-values.

In the context of administering the Neurotrophic peptide mixture, there was an elevation observed in the threshold for triggering the H-reflex of the gastrocnemius muscle, increasing from 5 [3; 9] mA to 6 [5.8; 18] mA (Fig. 4). Notably, there were no alterations noted in the latency period or power attributes of the H-reflex, nor in the parameters of the M-response, compared to their baseline values.

### Potentials P300

**Fig. 5:**
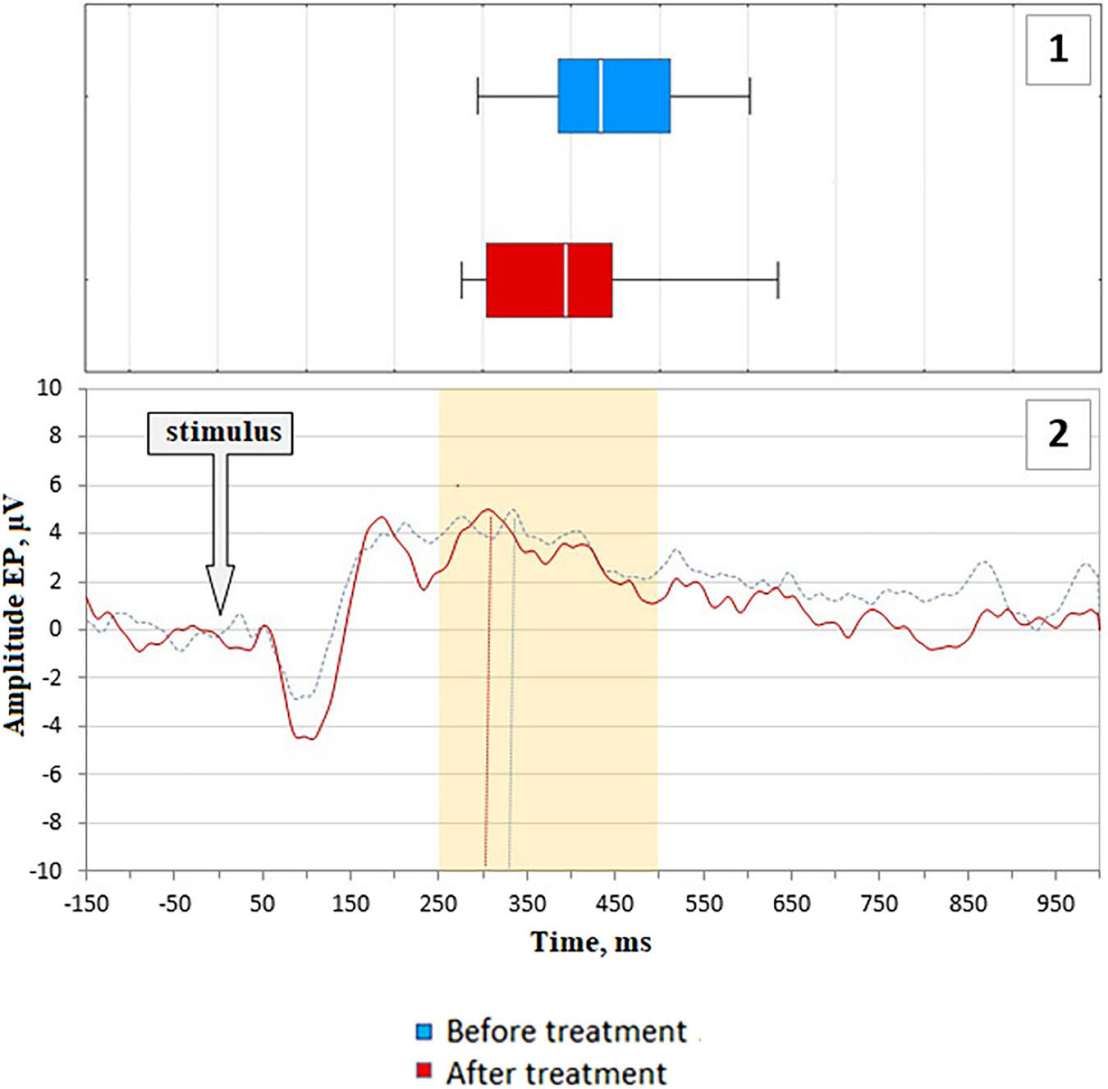
Analysis of P300 potentials. Latent period of the P3 component in the central parietal region (Pz). Endogenous evoked potentials averaged over the entire sample in the central parietal region (Pz). Blue bars represent values before treatment, red bars represent values after treatment. Data are shown as horizontal box plots with median (vertical line), interquartile range (box), minimum and maximum values (whiskers). Secondary/Exploratory outcomes: Descriptive statistics and effect sizes reported without inferential p-values.

Analysis of endogenous components revealed that in the studied group of patients with PD, there was a delay in the formation of the P3 wave (median 434 ms, interquartile range 386–512 ms in the temporal area of the neocortex) compared to the corresponding age norm of 390–410 ms. After the course of the Neurotrophic peptide mixture, a statistically significant increase in the latency period of the P3 component in the central-parietal lead was recorded, up to 394 [304; 446] ms (Fig. 5). There were no significant changes in the latency period of the N2 component and the amplitudes of the N2 and P3 components.

The delay in the formation of the P3 wave is a characteristic feature of several neurodegenerative diseases, particularly Alzheimer’s and Parkinson’s diseases. For patients with borderline PD and moderate cognitive impairments, an increase in the latency period of P3 has been described, with a reverse correlation between this parameter and the volume of gray matter in the globus pallidus, dysfunction of which leads to symptoms such as bradykinesia and rigidity^25^. Improvement in this parameter following the course of the Neurotrophic peptide mixture suggests that a more pronounced effect on the state of higher cortical functions can be expected in patients with increased risk of developing or progressing cognitive impairments.

### Correlation analysis of clinical and neurophysiological parameters after a course of treatment with the Neurotrophic peptide mixture

**Fig. 6:**
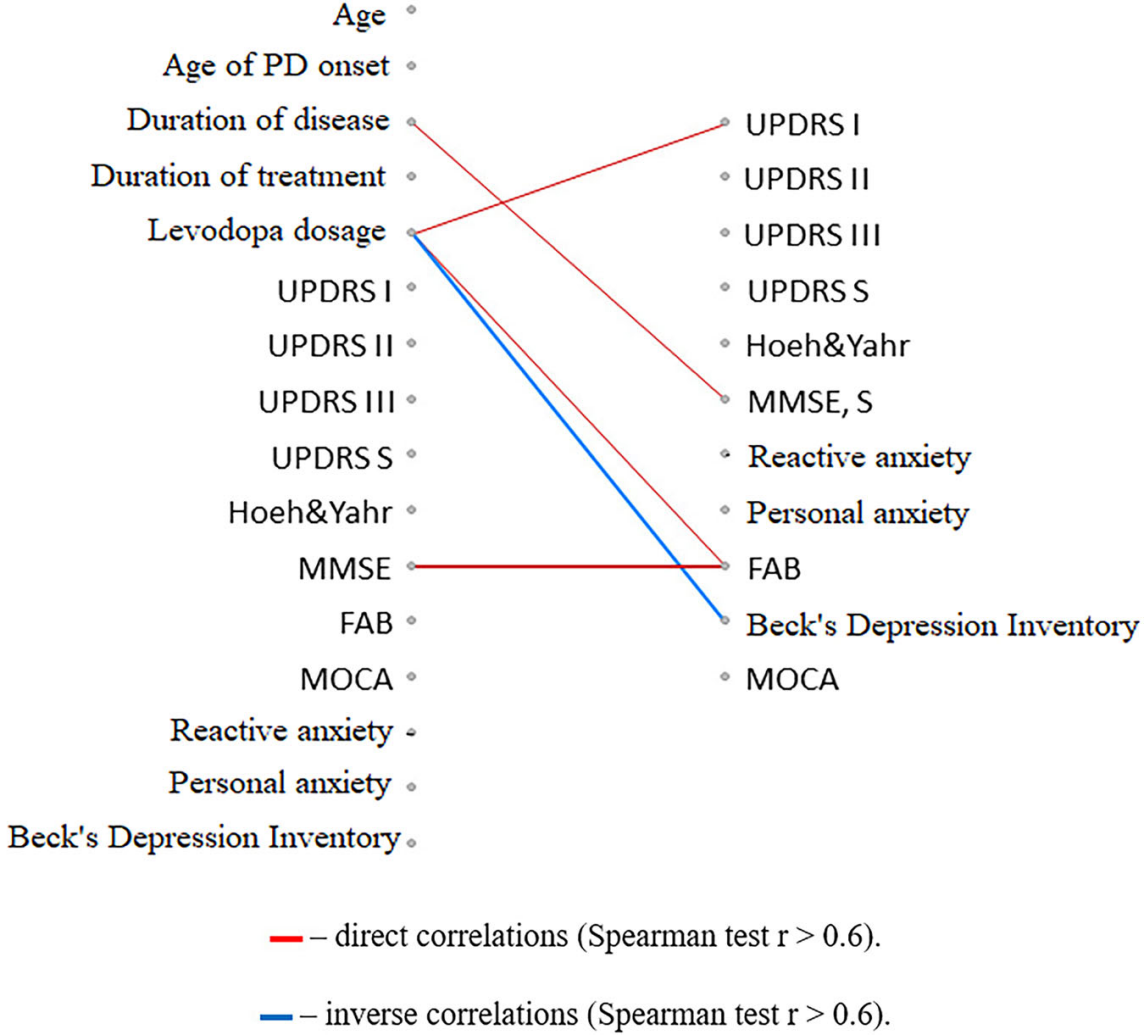
Correlation analysis of the relationships between changes in clinical scale indicators and the initial clinical characteristics of PD patients. The diagram represents the relationships between changes in clinical scale indicators after a course of the neurotrophic peptide mixture (right) and the initial clinical characteristics of patients with PD (left). Significant correlations (Spearman’s rank correlation coefficient *r* > 0.6) are shown. Red lines indicate direct correlations (positive associations), while blue lines represent inverse correlations (negative associations). Secondary/Exploratory correlations: Descriptive statistics reported without inferential p-values.

**Fig. 7:**
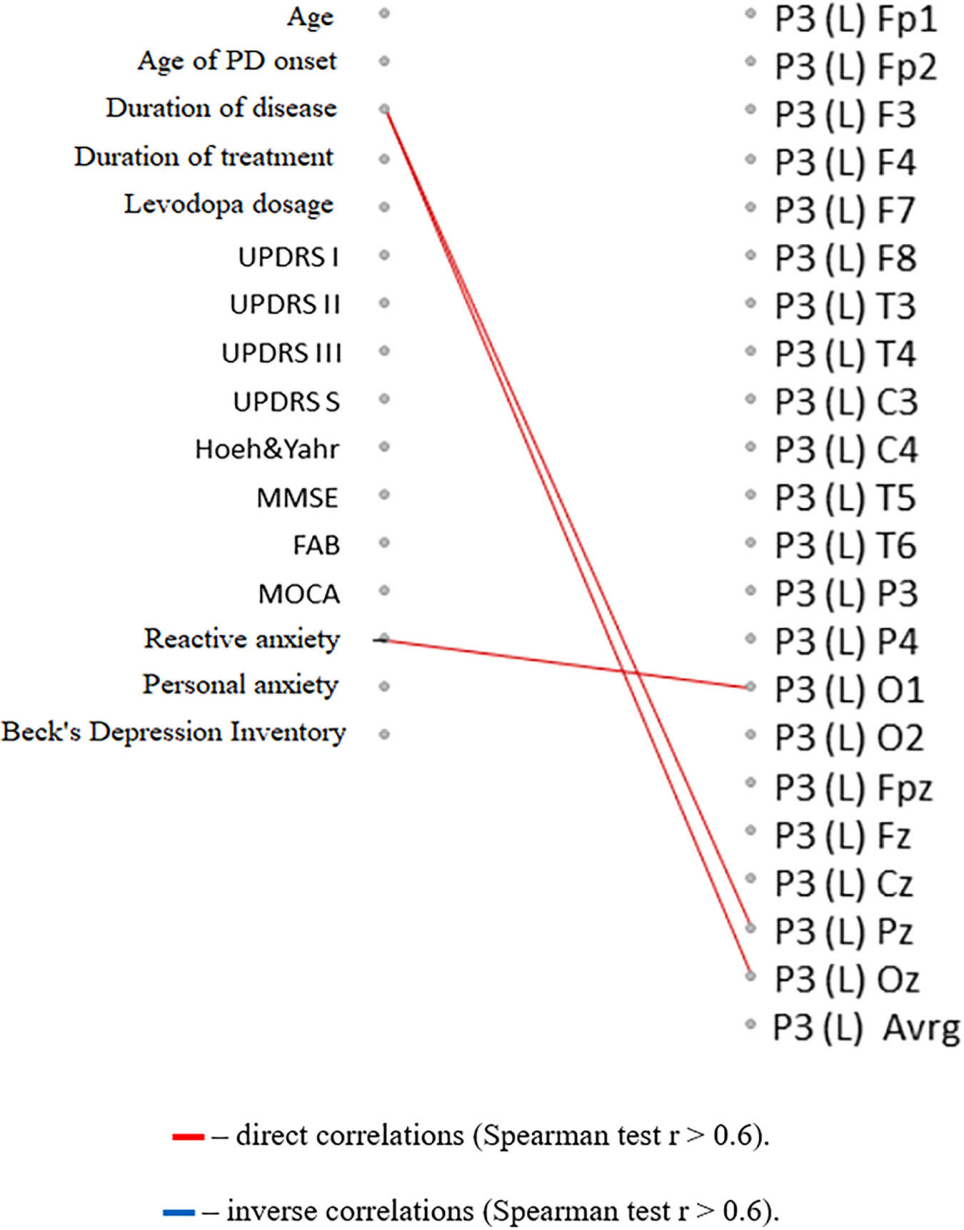
Correlation analysis of the relationship between changes in the latent period of the P3 component of the cognitive evoked potential of the brain with the initial clinical characteristics of PD patients. The diagram represents relationships between the latent period of the P3 component of the cognitive evoked potential of the brain after a course of the Neurotrophic peptide mixture (right) with the initial clinical characteristics of patients with PD (left). (S) – Sinister, (D) - Dexter. Significant correlations (Spearman’s rank correlation coefficient *r* > 0.6) are shown. Red lines indicate direct correlations (positive associations), while blue lines represent inverse correlations (negative associations). Secondary/Exploratory correlations: Descriptive statistics reported without inferential p-values.

**Fig. 8:**
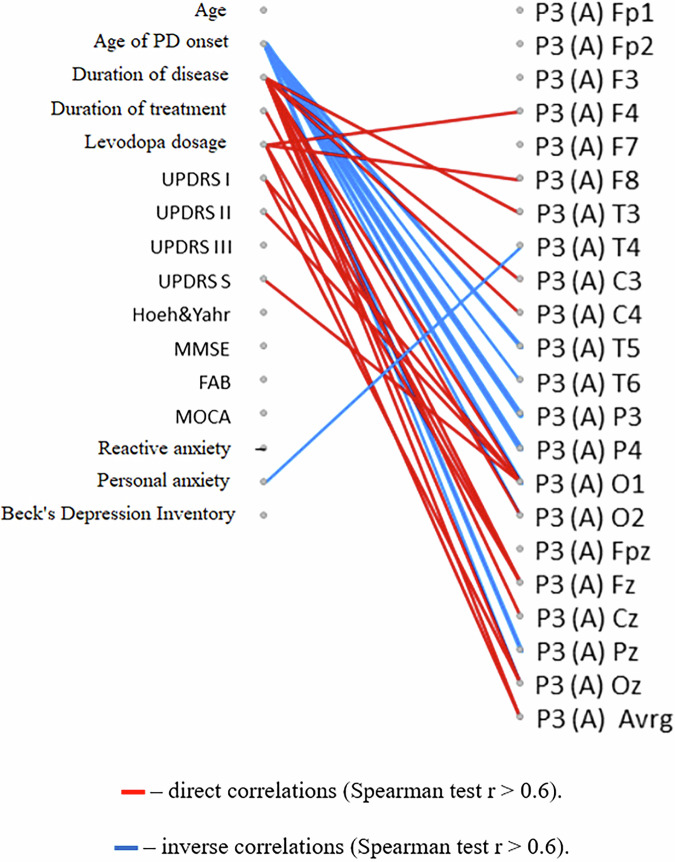
Correlation analysis of the relationship between changes in the amplitude of the P3 component of the cognitive evoked potential with the initial clinical characteristics of patients. The diagram represents relationships between changes in the amplitude of the P3 component of the cognitive evoked potential of the brain after a course of the Neurotrophic peptide mixture (right) with the initial clinical characteristics of patients with PD (left). (S) – Sinister, (D) - Dexter. Significant correlations (Spearman’s rank correlation coefficient *r* > 0.6) are shown. Red lines indicate direct correlations (positive associations), while blue lines represent inverse correlations (negative associations). Secondary/Exploratory correlations: Descriptive statistics reported without inferential p-values.

The degree of improvement in several non-motor characteristics (cognitive functions assessed by the FAB scale, amplitude of cognitive evoked potential P3, and signs of depression measured by the Beck scale) was greater in individuals who initially received a higher daily dose of levodopa (Figs. 6–8).

For indicators of cognitive functions (MMSE, P3 cognitive evoked potential amplitude), a similar relationship with the time of onset of the disease was also found: more pronounced positive effects of the Neurotrophic peptide mixture treatment were observed in patients with an earlier onset of PD and a longer duration of the disease (Figs. 6, 8).

**Fig. 9:**
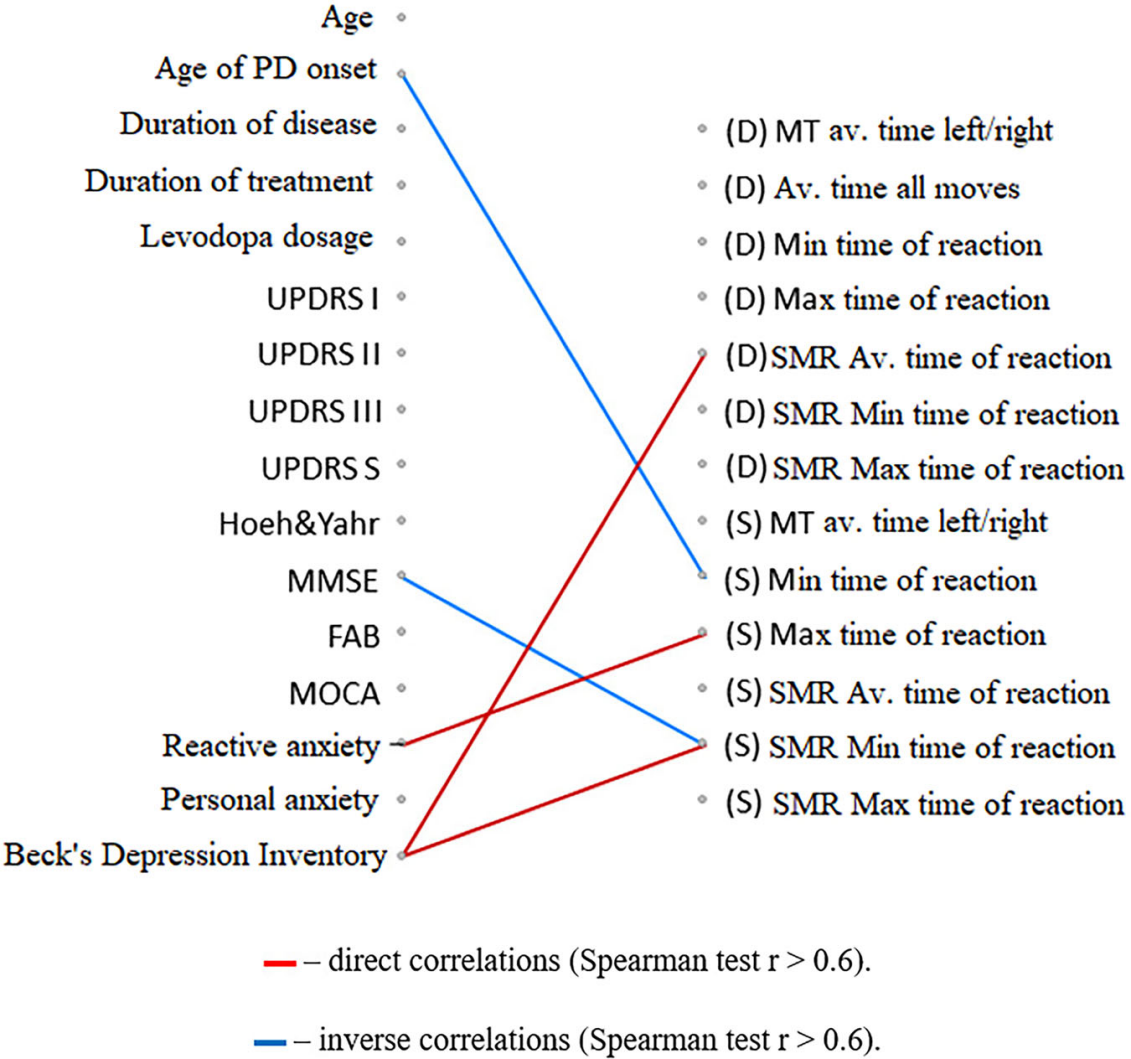
Correlation analysis of the relationships between changes in the temporal characteristics of motor and sensimotor reactions with the initial clinical characteristics of PD patients. The diagram represents relationships between changes in the temporal characteristics of motor reactions (motor tempo (MT), latent period of the sensorimotor reaction (SMR)) after a course of the Neurotrophic peptide mixture (right) with the initial clinical characteristics of patients with PD (left). (S) – Sinister, (D) - Dexter. Significant correlations (Spearman’s rank correlation coefficient *r* > 0.6) are shown. Red lines indicate direct correlations (positive associations), while blue lines represent inverse correlations (negative associations). Secondary/Exploratory correlations: Descriptive statistics reported without inferential *p*-values.

**Fig. 10:**
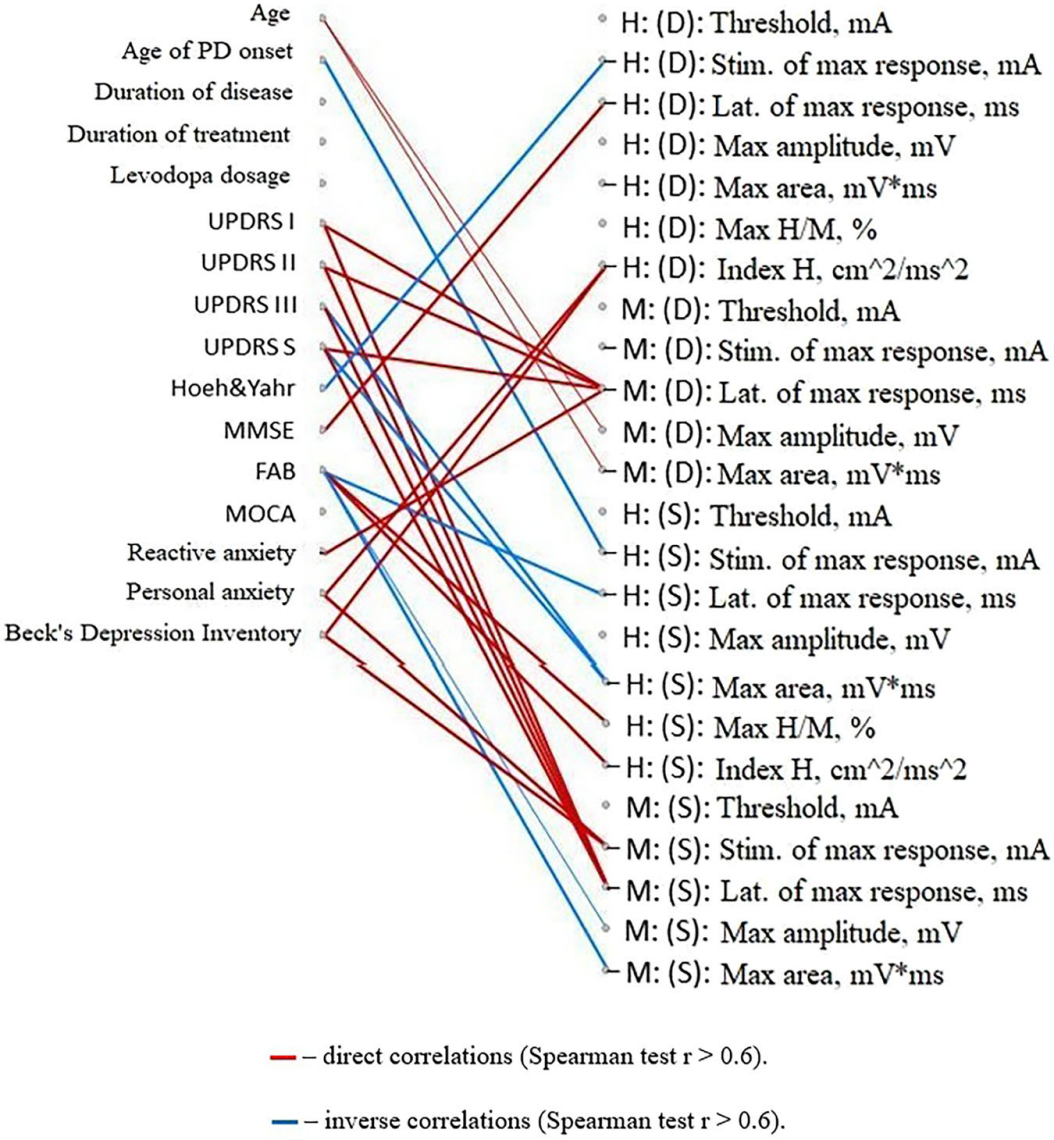
Correlation analysis of the relationships between changes in the electroneuromyographic characteristics of the H-reflex and M-response with the initial clinical characteristics of PD patients. The diagram represents relationships between the electroneuromyographic characteristics of the H-reflex and M-response after a course of the Neurotrophic peptide mixture (right) with the initial clinical characteristics of patients with PD (left). (S) – Sinister, (D) - Dexter. Significant correlations (Spearman’s rank correlation coefficient *r* > 0.6) are shown. Red lines indicate direct correlations (positive associations), while blue lines represent inverse correlations (negative associations). Secondary/Exploratory correlations: Descriptive statistics reported without inferential *p*-values.

At the same time, a more pronounced reduction in reaction time was characteristic of individuals with lower initial ratings of PD severity on the UPDRS scale and lower levels of anxiety. Such correlation relationships are found both when directly assessing the time of motor reactions in the Motor tempo and Sensorimotor reaction tests, and when determining the latent periods of the H-reflex and M-response (Figs. 9, 10).

The use of the Neurotrophic peptide mixture in the treatment course improves the general condition of patients with Parkinson’s disease, as evidenced by a significant decrease in the quantitative indicators of all UPDRS subscales, attenuation of affective disorders such as anxiety and depression, and improvement in cognitive function. Against the background of the Neurotrophic peptide mixture administration, dynamic characteristics of simple motor reactions improve, and the threshold for the emergence of the H-reflex increases, indicating facilitation of supraspinal control processes over the activity of motor neurons. In general, the results show a strong tendency to improvement in different parameters.

### Mitochondrial dysfunction before and after treatment with the Neurotrophic peptide mixture in patients with Parkinson’s disease

**Fig. 11:**
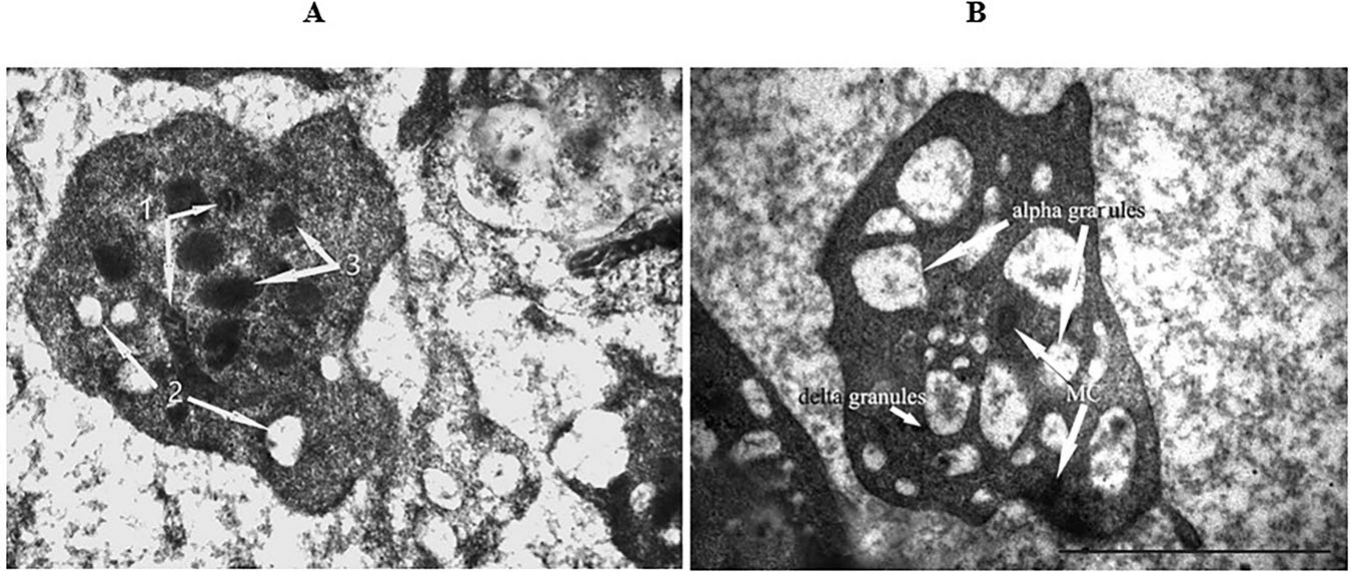
Platelets ultrastructure analysis. The electron microscopy images (1 μm scale) represent the ultrastructure of platelets of individuals from the control group (**A**) and patients with Parkinson’s disease (**B**). 1 – Mitochondria. 2 – Alpha granules. 3 – Delta granules.

The conducted studies revealed significant differences in the structural organization of platelets in individuals with PD examined before the start of the standard treatment process. Electron microscopic and morphometric study of platelets in patients with PD revealed that platelets were mainly represented by senescent cells. They had a reduced number of dense δ granules, which was 5.1 ± 0.4 per one cell (in healthy individuals - 8.4 ± 0.5 units per one cell, *p* < 0.05). However, the number of transparent α-granules turned out to be increased from 5.3 ± 0.2 up to 10.3 ± 0.6 per cell. In addition, the latter often had large sizes (>100 nm) and contained a significant amount of protein debris (Fig. 11).

The single mitochondria that were found in the cells (2–4 per cell) were structurally quite preserved and had an average diameter of 0.48 ± 0.02 µm, not significantly different from those determined in the control group (0.41 ± 0.04 μm).

Significant abnormalities were found in the ultrastructure of mitochondria in platelets of patients. In addition to increased mitochondrial membrane permeability (outer and/or inner, crystas-forming), the formation of septated organelles was observed. The mechanisms and causes of their formation are still unexplored. It is assumed that septated mitochondria can accumulate Са^2+^^26^. Since it is known that the accumulation of Са^2+^ and the synthesis of ATP are alternative processes associated with the transfer of electrons, an increase in the Ca-accumulating ability of mitochondria may indicate a decrease in the synthesis of ATP in them. In addition, the appearance of septated mitochondria may indicate partial cell necrosis. These ultrastructural changes are evidence of the formation of mitochondrial dysfunction in platelets.

The use of the Neurotrophic peptide mixture in the complex therapy of patients with CP was accompaniedby reliable dynamics of the morphofunctional state of platelets (Table 1). The use of the Neurotrophic peptide mixture led to a significant (by 45%) increase in the number of δ-granules. No significant changes in the number of α granules were detected; only a tendency to its decrease was observed. An increase in the number of δ-granules indicates an increase in serotonin reserves. Probably, one of the possible mechanisms of the neuroprotective effect of the Neurotrophic peptide mixture is realized in this way. In addition, the Ca^2+^ -accumulating capacity in thrombocytes is relatively reduced, which should contribute to the improvement of ATP synthesis.

**Table 1:** Characteristics of the ultrastructure of platelets in patients with Parkinson’s disease before and after treatment with the Neurotrophic peptide mixture

Groups of patients	δ-granules, pcs / cell	α- granules, pcs / cell
Control group	8,4 ± 0,5	5,3 ± 0,2
PD patients before treatment	5,1 ± 0,4	10,3 ± 0,6
PD patients after treatment with neurotrophic peptide mixture	7,4 ± 0,3	9,2 ± 0,4

Secondary/Exploratory outcomes: Descriptive statistics reported without inferential *p*-values.

Despite the small number of mitochondria in platelets, a slight increase in their number was observed - from 2 to 4 organelles before treatment to 5–7 organelles after treatment with the Neurotrophic peptide mixture, which, in combination with the absence of detection of septated organelles, may indicate a decrease in the severity of mitochondrial dysfunction.

Thus, treatment with the Neurotrophic peptide mixture improves the morphofunctional state of platelets in patients with PD, it increases serotonin reserves, reduces Ca^2+^ -accumulating capacity, reduces the manifestations of mitochondrial dysfunction, and exhibits mild antithrombotic properties.

### Effect of the Neurotrophic peptide mixture treatment on PARKIN gene expression in patients with Parkinson’s disease

For the whole group of tested individuals, taken together, no correlation of the expression of the measured genes was shown using the Spearman test. At the same time, when dividing by sex, multidirectional differences in gene expression before and after therapy with the drug Neurotrophic peptide mixture were found (Table 2).

**Table 2:** Spearman t-test results for correlation and PARKIN gene expression

	BDNF	DJ-1	PINK1	TNFalfa
Men	0.025063	0.16	0.57	0.16
Women	0.046	0.17	0.17	0.115

Secondary/Exploratory outcomes: Descriptive statistics reported without inferential *p*-values.

**Fig. 12:**
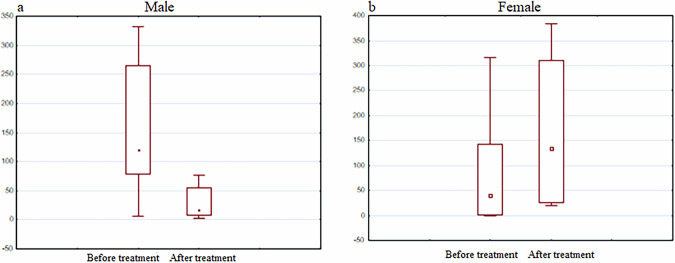
BDNF gene expression analysis. The diagrams represent BDNF gene expression in men (**a**) and women (**b**) before and after the treatment with the Neurotrophic peptide mixture. The analysis was performed using Wilcoxon Matched Pairs Test. Data are shown as box plots with median (dot in men, square in women), interquartile range (box), minimum and maximum values (whiskers). Secondary/Exploratory outcomes: Descriptive statistics reported without inferential p-values.

In particular, the group of men was characterized by a decrease in the expression of the BDNF gene, and in women the expression increased after the use of the drug (Fig. 12).

**Fig. 13:**
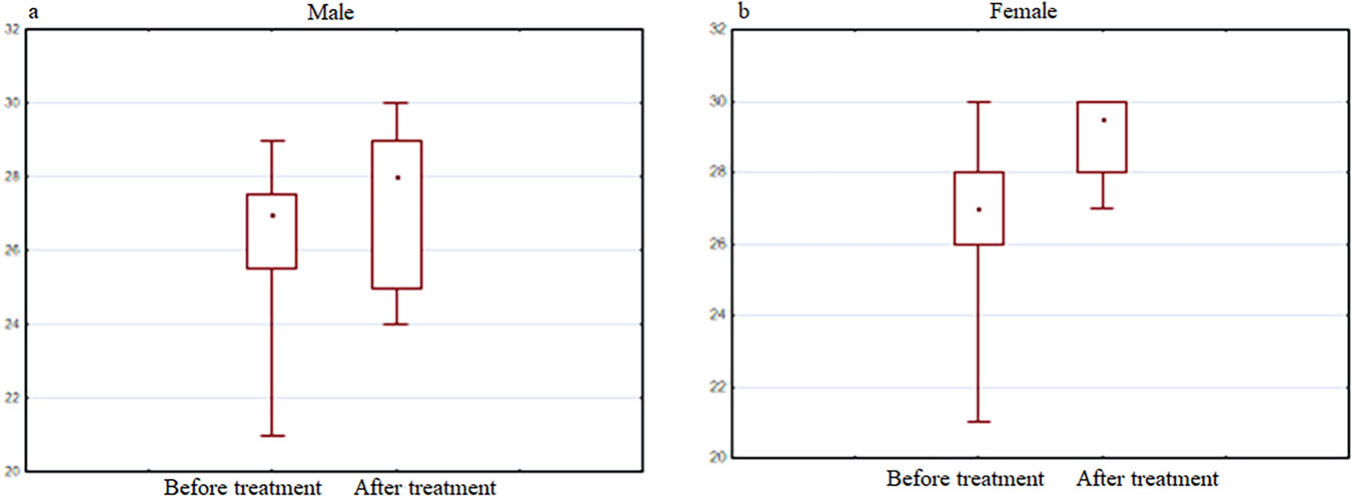
MMSE index. The diagrams represent MMSE index in men (**a**) and women (**b**) before and after the treatment with the Neurotrophic peptide mixture. The analysis was performed using Wilcoxon Matched Pairs Test. Data are shown as box plots with median (dot), interquartile range (box), minimum and maximum values (whiskers). Secondary/Exploratory outcomes: Descriptive statistics reported without inferential *p*-values.

It is important to note that when comparing the results of gene expression with cognitive parameters, an increase in BDNF in women (but not in men) was also accompanied by an increase in the MMSE score (Fig. 13).

**Fig. 14:**
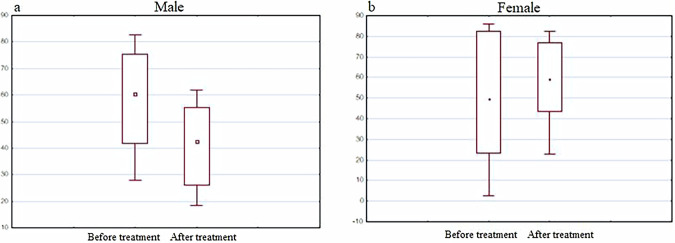
DJ-1 gene expression analysis. The diagrams represent DJ-1 gene expression in men (**a**) and women (**b**) before and after the treatment with the Neurotrophic peptide mixture. The analysis was performed using Wilcoxon Matched Pairs Test. Data are shown as box plots with median (square in men, dot in women), interquartile range (box), minimum and maximum values (whiskers). Secondary/Exploratory outcomes: Descriptive statistics reported without inferential *p*-values.

**Fig. 15:**
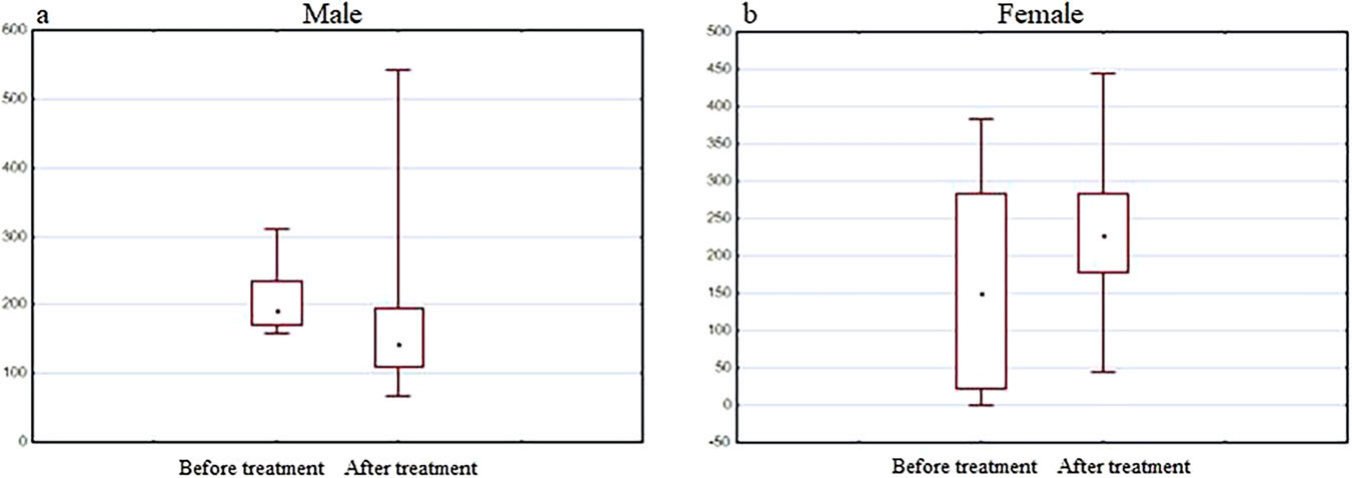
PINK1 gene expression analysis. The diagrams represent PINK1 gene expression in men (**a**) and women (**b**) before and after the treatment with the Neurotrophic peptide mixture. The analysis was performed using Wilcoxon Matched Pairs Test. Data are shown as box plots with median (dot), interquartile range (box), minimum and maximum values (whiskers). Secondary/Exploratory outcomes: Descriptive statistics reported without inferential *p*-values.

**Fig. 16:**
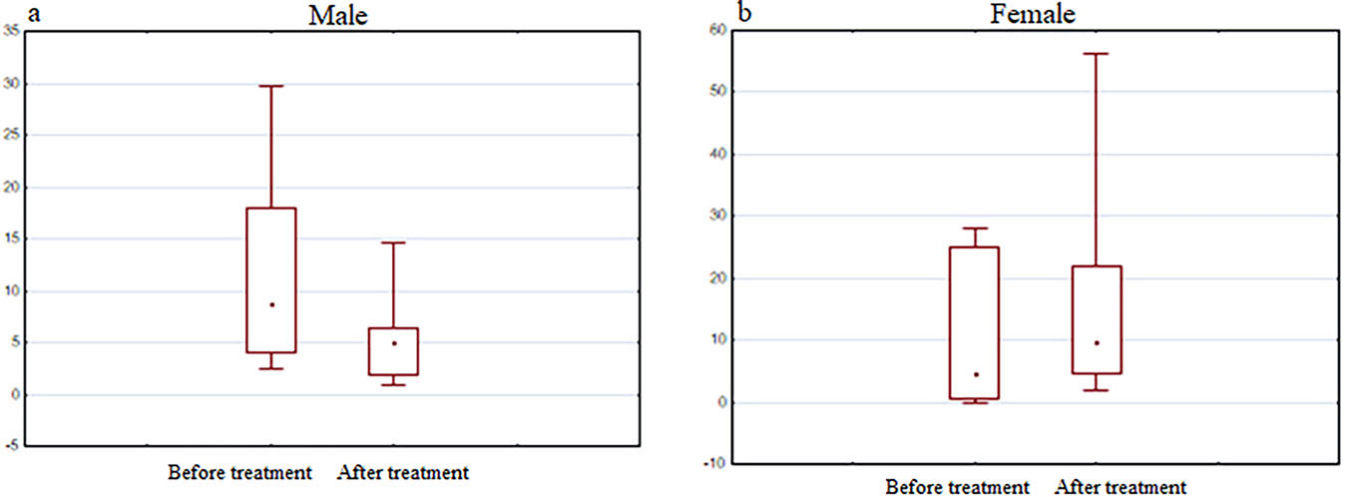
TNFalfa gene expression analysis. The diagrams represent TNFalfa gene expression in men (**a**) and women (**b**) before and after the treatment with the Neurotrophic peptide mixture. The analysis was performed using Wilcoxon Matched Pairs Test. Data are shown as box plots with median (dot), interquartile range (box), minimum and maximum values (whiskers). Secondary/Exploratory outcomes: Descriptive statistics reported without inferential p-values.

No statistically significant difference was observed in expression of other three genes (DJ-1, PINK1, TNFalfa) before and after treatment with the Neurotrophic peptide mixture (Figs. 14–16).

The correlation analysis shows higher values between MMSE and PINK1 expression, FAB a and DJ1, FAB a and PINK1, MOCA a and DJ1, MOCA a and PINK1, MOCA and PINK1 for men (Table 3).

**Table 3:** Correlation matrix on the interdependence of various parameters and the expression of measured genes for men

	BDNF (before)	BDNF (after)	DJ1 (before)	DJ1 (after)	PINK1 (before)	PINK1 (after)	TNFalfa (before)	TNFalfa (after)
UPDRS I a	−0.20	−0.10	−0.17	−0.35	−0.40	−0.27	−0.01	−0.37
UPDRS I	0.33	−0.28	0.21	0.00	0.33	−0.18	0.46	−0.39
UPDRS II a	−0.30	−0.04	−0.33	−0.05	−0.19	0.00	−0.08	−0.20
UPDRS II	0.30	−0.05	0.28	−0.11	0.22	−0.33	0.42	−0.25
UPDRS III a	−0.11	−0.25	−0.29	−0.28	0.08	−0.13	−0.05	−0.36
UPDRS III	0.35	−0.20	0.33	−0.39	0.20	−0.47	0.41	−0.31
UPDRS S a	−0.33	−0.02	−0.43	−0.33	−0.21	−0.19	−0.21	−0.33
UPDRS S	0.29	−0.04	0.26	−0.11	0.20	−0.26	0.38	−0.11
Hoeh Yahr a	−0.11	0.16	−0.22	−0.16	0.03	0.02	−0.22	0.26
Hoeh Yahr	−0.11	0.16	−0.22	−0.16	0.03	0.02	−0.22	0.26
MMSE. S a	0.28	0.22	0.43	−0.22	−0.01	−0.38	0.10	−0.03
MMSE. S	0.15	0.31	0.27	−0.53	0.01	−0.74	−0.01	−0.43
RA Spielb a	−0.06	0.06	0.11	0.35	0.11	0.08	0.22	−0.07
RA Spielb	0.11	−0.38	0.06	−0.10	0.05	0.01	0.28	−0.06
PA Spielb a	−0.48	−0.05	−0.50	0.02	−0.24	0.10	−0.24	−0.31
PA Spielb	−0.38	−0.07	−0.38	−0.02	−0.07	0.00	−0.17	−0.36
FAB a	−0.06	0.04	0.06	−0.87	−0.57	−0.73	−0.14	−0.46
FAB	0.50	0.10	0.63	−0.28	0.30	−0.60	0.35	−0.33
Beka A	−0.22	−0.17	−0.19	0.18	−0.07	0.19	0.07	−0.08
Beka	−0.19	−0.12	−0.13	0.18	0.00	0.12	0.10	−0.14
MOCA a	−0.22	0.32	−0.06	−0.80	−0.51	−0.79	−0.28	−0.48
MOCA	0.04	0.18	0.21	−0.68	−0.23	−0.82	0.00	−0.60
Age	0.11	−0.23	−0.06	0.51	0.23	0.66	0.12	0.56

Spearman t-test was used for analysis, *р* < 0.05 considered significant and labeled with asterisks.

Secondary/Exploratory outcomes: Descriptive statistics reported without inferential *p*-values.

For women, the correlation analysis shows strong values of clinically central endpoints UPDRS III and UPDRS S with almost all studied genes expression rates (Table 4).

**Table 4:** Correlation matrix on the interdependence of various parameters and the expression of measured genes for women

	BDNF (before)	BDNF (after)	DJ1 (before)	DJ1 (after)	PINK1 (before)	PINK1 (after)	TNFalfa (before)	TNFalfa (after)
UPDRS II	−0.70	−0.70	−0.64	−0.49	−0.64	−0.61	−0.75	−0.70
UPDRS III a	−0.97*	−0.88*	−0.88*	−0.77	−0.88*	−0.97*	−0.97*	−0.88*
UPDRS III	−0.89*	−0.83*	−0.77	−0.60	−0.77	−0.94*	−0.94*	−0.83*
UPDRS S a	−0.87*	−0.78	−0.72	−0.58	−0.72	−0.87	−0.93	−0.78
UPDRS I a	−0.27	−0.03	−0.03	0.14	−0.03	−0.44	−0.27	−0.03
UPDRS I	−0.39	−0.13	−0.13	−0.13	−0.13	−0.39	−0.39	−0.13
UPDRS II a	−0.60	−0.60	−0.54	−0.37	−0.54	−0.54	−0.66	−0.60
UPDRS S	−0.89*	−0.83*	−0.77	−0.66	−0.77	−0.83*	−0.94*	−0.83*
Hoeh Yahr a	-	-	-	-	-	-	-	-
Hoeh Yahr	-	-	-	-	-	-	-	-
MMSE. S a	−0.20	−0.41	−0.26	−0.12	−0.26	−0.06	−0.35	−0.41
MMSE. S	−0.39	−0.70	−0.58	−0.33	−0.58	−0.39	−0.52	−0.70
RA Spielb a	−0.58	−0.52	−0.64	−0.64	−0.64	−0.39	−0.46	−0.52
RA Spielb	−0.17	−0.09	−0.23	−0.29	−0.23	0.00	−0.03	−0.09
PA Spielb a	−0.81	−0.49	−0.67	−0.75	−0.67	−0.81	−0.64	−0.49
PA Spielb	0.18	0.53	0.26	0.18	0.26	0.09	0.44	0.53
FAB a	0.20	−0.20	0.03	0.20	0.03	0.31	−0.03	−0.20
FAB	0.06	−0.34	−0.19	−0.19	−0.19	0.22	−0.09	−0.34
Beka A	−0.06	0.23	−0.06	−0.12	−0.06	−0.20	0.23	0.23
Beka	−0.14	0.31	0.09	−0.09	0.09	−0.26	0.09	0.31
MOCA a	0.09	−0.26	−0.03	0.09	−0.03	0.26	−0.14	−0.26
MOCA	−0.14	−0.54	−0.31	−0.26	−0.31	0.09	−0.37	−0.54
Age	−0.09	−0.17	−0.03	0.06	−0.03	0.03	−0.23	−0.17

Spearman t-test was used for analysis, *р* < 0.05 considered significant and labeled with asterisks.

Secondary/Exploratory outcomes: Descriptive statistics reported without inferential *p*-values.

### Effect of the Neurotrophic peptide mixture treatment on the state of the prooxidant-antioxidant system in patients with Parkinson’s disease

The intensification of prooxidant processes in blood plasma and erythrocytes of patients with moderate PD stages was registered. It was established a significant rise (by 40%) in the content of the secondary products of lipid peroxidation in blood plasma as well as a marked increase (by 50%) of the H_2_O_2_ formation in erythrocytes of patients with PD compared to healthy participants (Tables 5 and 6). Our assessment of the antioxidant protective effectiveness was largely based on the changes in activity of antioxidant enzymes – SOD and catalase, which represent the first line of such defense, providing detoxification of reactive oxygen metabolites^27^. In patients with PD, we have found an increase in the activities of SOD and catalase by 21% and 69%, respectively in comparison with healthy subjects. In addition, we analyzed the glutathione cycle, which is one of the main intracellular mechanisms for maintaining redox homeostasis^28^. In our study, the concentration of GSH was significantly decreased (by 58%) in erythrocytes of PD patients as well as GPx activity though this change was not statistically significant (by 15%) compared to healthy controls. We found that CBL administration has moderate antioxidant effects in PD patients by decreasing the TBARS accumulation (by 17%) in blood plasma as well as H_2_O_2_ content in erythrocytes (by 12%) compared with the CBL-untreated patients. These effects of CBL were accompanied by a decrease in the activities of SOD (by 11%) and catalase (by 20%) in blood of patients with PD compared with the CBL-untreated ones. In CBL-treated patients, there was registered an increase in the GSH concentration (by 30%) and GPx activity (by 15%) in erythrocytes compared with PD patients though this change was not statistically significant compared to the CBL-untreated patients (Tables 5 and 6).

**Table 5:** Effect of the Neurotrophic peptide mixture administration on oxidative and anti-oxidative status in plasma of PD patients

Parameters	Healthy Control	Patients with Parkinson’s disease	Patients with Parkinson’s disease after treatment
TBARS, μM/ml	5.01 ± 0.85	6.99 ± 1.93	5.79 ± 1.95
SOD, U/ml	11.99 ± 1.32	13.22 ± 1.67	11.73 ± 2.23
Catalase, μM/min/ml	3.24 ± 0.61	7.16 ± 1.87	5.75 ± 1.41

Values are shown as mean ± SD (Standard deviation). The data were analyzed for statistical significance using ANOVA followed by the Bonferroni post hoc test.

Secondary/Exploratory outcomes: Descriptive statistics reported without inferential p-values.

**Table 6:** Effect of the Neurotrophic peptide mixture administration on oxidative and anti-oxidative status in erythrocytes of PD patients

Parameters	Healthy Control	Patients with Parkinson’s disease	Patients with Parkinson’s Disease after treatment
H2O2, µM /mg Hb	9.26 ± 0.65	13.83 ± 1.77	12.22 ± 2.35
GSH, μM/mg Hb	9.25 ± 1.44	3.91 ± 2.06	5.09 ± 2.48
GPx, nM NADPH/min/mgHb	10.39 ± 1.64	8.84 ± 1.70	10.11 ± 1.96

Values are shown as mean ± SD (Standard deviation). The data were analyzed for statistical significance using ANOVA followed by the Bonferroni post hoc test.

Secondary/Exploratory outcomes: Descriptive statistics reported without inferential *p*-values.

### UPDRS score change prediction using multiple machine learning models

Among the models, GBMs demonstrated the highest predictive accuracy, achieving an RMSE of 18.59, MSE of 345.82, and MAE of 15.39. The top-performing RF model exhibited slightly higher RMSE (21.99) and MAE (17.78), underscoring the superior capability of GBMs to capture complex patterns in the data.

**Fig. 17:**
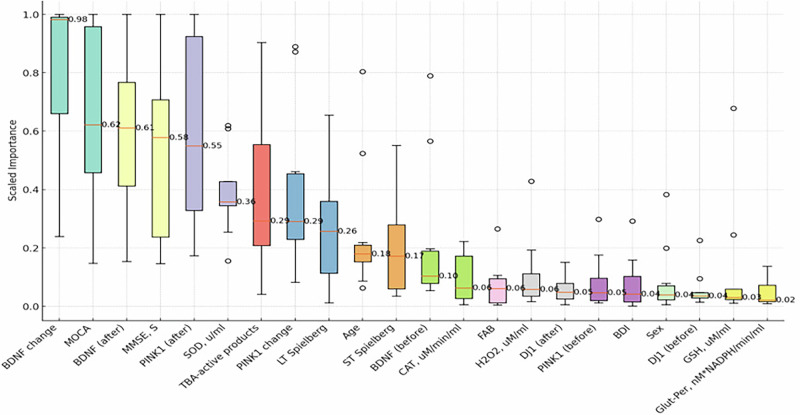
Boxplot of feature importances sorted by decreasing median. The figure presents normalized importance scores of clinical, biochemical, genetic, and neuropsychological parameters used in the predictive model. The y-axis shows scaled importance (0–1), and the x-axis lists the included variables. The most influential predictors were changes in BDNF levels, MoCA scores, baseline BDNF concentration, MMSE, and PINK1, followed by oxidative stress markers (SOD activity, TBA-reactive products) and additional clinical/neuropsychological measures. Boxplots reflect variability in importance estimates across repeated model iterations, with outliers shown as dots.

The boxplot of feature importance across the top 10 models provided valuable insights into which factors most significantly influenced the prediction of UPDRS score changes. Below are the most impactful features, sorted by median scaled importance: Fig. 17

- BDNF Change (Median Importance: 0.98): Changes in Brain-Derived Neurotrophic Factor (BDNF) were the most influential predictor of UPDRS score change. BDNF’s high importance suggests that neurotrophic support may play a central role in the Neurotrophic peptide mixture’s therapeutic effects, potentially enhancing neuroplasticity and recovery.
- MOCA (Median Importance: 0.62): The Montreal Cognitive Assessment (MOCA), a measure of cognitive function, was ranked as a highly predictive feature, highlighting the relevance of cognitive improvement as a response indicator for the Neurotrophic peptide mixture therapy.
- BDNF (after treatment) (Median Importance: 0.61): Post-treatment levels of BDNF were also significant, indicating that maintaining or boosting neurotrophic factors is associated with positive therapeutic outcomes in PD patients.
- MMSE, S (Median Importance: 0.58): The Mini-Mental State Examination (MMSE) score also emerged as a key predictor, reinforcing the importance of cognitive assessments in evaluating the Neurotrophic peptide mixture’s efficiency.
- PINK1 (after treatment) (Median Importance: 0.55): PINK1, a mitochondrial health marker, was another significant predictor, suggesting that the Neurotrophic peptide mixture’s potential benefits may extend to cellular and mitochondrial health.

The boxplot analysis revealed that features related to neurotrophic support (BDNF) and cognitive function (MOCA, MMSE) showed the most consistent importance across models, as evidenced by their high median importance values and narrow interquartile ranges. In contrast, features with wider variability, such as TBA-active products and Age, had broader interquartile ranges, indicating that their contribution to predictive accuracy may depend on model-specific interactions or particular patient subgroups.

## Discussion

The findings of this exploratory study suggest that the Neurotrophic peptide mixture has a multifaceted impact on various aspects of PD pathology, particularly regarding platelet ultrastructure, gene expression, neurophysiological parameters, and oxidative stress markers. These findings align with existing literature, which underscores the neuroprotective and neurorestorative properties of the Neurotrophic peptide mixture.

Improvement in motor functions, particularly in the Motor tempo test, was more pronounced in patients with predominantly right-sided symptoms of PD. This asymmetry in response could be related to the lateralization of the pathological process. Additionally, the elevation in the threshold for triggering the H-reflex suggests modifications in spinal cord excitability, which could translate to improvements in motor function. The study reported statistically significant enhancements in cognitive abilities, as measured by the MMSE, FAB, and MoCA scales. Additionally, there was a notable reduction in depression scores (BDI) and reactive anxiety (Spielberger-Hanin scale), although personal anxiety levels remained unchanged. These findings highlight the potential of the Neurotrophic peptide mixture in ameliorating both motor and non-motor symptoms of PD. The improvements in motor function, cognitive abilities, and emotional well-being observed in this study suggest that the Neurotrophic peptide mixture has a broad therapeutic potential.

The study also observed significant neurophysiological improvements post-Neurotrophic peptide mixture treatment. The latency period of the P3 component, a marker of cognitive processing, was significantly reduced. This improvement in P3 latency indicates enhanced cortical function and suggests that the Neurotrophic peptide mixture may have beneficial effects on cognitive processing speed and efficiency in PD patients. These neurophysiological benefits align with the observed cognitive improvements, further supporting the potential of the Neurotrophic peptide mixture in mitigating cognitive decline in PD.

In PD patients, platelets exhibited several signs of senescence, including a reduction in dense δ-granules and an increase in α-granules^29^. The number of δ-granules per cell was significantly lower in PD patients (5.1 ± 0.4) compared to healthy individuals (8.4 ± 0.5). The administration of the Neurotrophic peptide mixture significantly increased the number of δ-granules in platelets by 45%, indicating enhanced serotonin reserves, which could contribute to the neuroprotective effects observed clinically. This increase in δ-granules likely reflects a normalization of platelet storage function, which is crucial given the role of serotonin in mood and neuroprotection. Despite no significant change in the number of α-granules, the trend towards a decrease might indicate an alteration in platelet activation status.

Additionally, the Neurotrophic peptide mixture reduced the calcium-accumulating capacity of platelets, potentially improving ATP synthesis and overall platelet function. This reduction in calcium accumulation is crucial as it indicates a shift away from the pathological state observed in untreated PD platelets. The observed improvements in platelet ultrastructure and function may translate to better overall cellular health, reflecting the systemic benefits of the Neurotrophic peptide mixture treatment beyond its direct neural effects. The enhancement in serotonin reserves and mitochondrial function underscores the multifaceted role of the Neurotrophic peptide mixture in addressing both neurological and peripheral manifestations of PD.

The Neurotrophic peptide mixture treatment appears to modulate BDNF gene expression in a sex-specific manner in patients with Parkinson’s disease, with women showing an increase and men showing a decrease in expression levels. This modulation is accompanied by improved cognitive function in women, highlighting the potential of the Neurotrophic peptide mixture as a therapeutic option that may benefit female PD patients more significantly. The stable expression of DJ-1, PINK1, and TNFα genes suggests that the primary neuroprotective mechanisms of the Neurotrophic peptide mixture in PD may involve pathways other than those mediated by these genes. These findings emphasize the need for further research to explore the underlying mechanisms of sex differences in PD treatment responses and to optimize therapeutic strategies accordingly.

The excessive production and release of ROS in PD occurs as a result of the combined action of mitochondrial dysfunction, dopamine autooxidation, and neuroinflammation^30^. In present study, the Neurotrophic peptide mixture demonstrated a moderate antioxidant effect, reducing the levels of lipid peroxidation products and H2O2 in erythrocytes. This reduction was accompanied by a decrease in SOD and catalase activities, and an increase in GSH concentration and GPx activity. These changes indicate a restoration of redox balance and a reduction in oxidative stress, which are crucial in the pathology of PD. One of the most promising effects of CBL on OS developing in blood and tissues in PD could be its ability to decrease intracellular α-synuclein aggregation in neurons, mimicking the action of classical NTFs and growth factors as well as of NTFs belonged to a novel family CDNF/MANF^31^. The ability of the Neurotrophic peptide mixture to modulate the antioxidant system suggests its potential role in protecting neuronal cells from oxidative damage and subsequent apoptosis.

The findings underscore the central role of cognitive and neurotrophic markers in predicting UPDRS score changes as an indicator of the Neurotrophic peptide mixture efficacy. The high importance of BDNF aligns with previous research on neuroprotective therapies, where neurotrophic support has been linked to improved neuronal survival and plasticity. The inclusion of mitochondrial markers, such as PINK1, suggests that the Neurotrophic peptide mixture may exert beneficial effects at the cellular level, addressing mitochondrial dysfunction—a known factor in PD pathology.

The boxplot analysis provided further clarity on the stability and reliability of each feature’s importance. Features with high median importance and narrow interquartile ranges, like BDNF and MOCA, indicate consistent predictive power across models, making them robust indicators of therapeutic response. The broader variability observed in other features, such as oxidative stress markers and demographic factors, suggests that while they may contribute to specific aspects of disease progression, they are less central to predicting therapeutic efficacy. The superior performance of GBMs in capturing non-linear relationships reinforces the value of using advanced machine learning techniques to analyze complex clinical datasets. These findings support the potential of the Neurotrophic peptide mixture as a multifaceted therapeutic option, with the capacity to improve cognitive and neurotrophic parameters in PD patients. The results of the present study indicate an antioxidant action of the Neurotrophic peptide mixture in patients with moderate stages of Parkinson’s disease. This action is mainly based on the decrease of the prooxidant events in peripheral blood of PD patients as well as on the prevention of thiol redox defects and reducing an imbalance in oxidant–antioxidant status.

Overall, statistically significant improvements and consistent correlations were observed in the key clinical endpoints (UPDRS Part III and UPDRS S), while secondary and exploratory outcomes demonstrated patterns suggestive of mechanistic links that warrant confirmation in larger studies.

Interpretation of its results is limited by several factors, including the small sample size, lack of a control group receiving placebo in addition to standard therapy, and a relatively short duration of the observation period. Since the study is preliminary, exploratory in nature and aimed at collecting primary data on the response to the drug, only one group of patients was formed, which allows identifying primary trends and assessing the feasibility of future larger studies. Future research should focus on larger cohorts and longer treatment durations to confirm these findings and to explore the long-term effects of neurotrophic peptide mixture on gene expression and cognitive function in PD patients. Particular attention should be paid to investigating sex differences in response to the drug in future studies.

The results of this exploratory study provide a preliminary rationale for investigating the Neurotrophic peptide mixture as an adjunct therapy in Parkinson’s disease. During the treatment period, changes were observed in platelet ultrastructure, gene expression, neurophysiological parameters, and oxidative stress markers. These findings, along with shifts in cognitive and neurotrophic markers, may suggest a multidimensional biological response.

However, these observations must be interpreted with extreme caution. Given the limited sample size and the absence of a control group, no causal inferences can be drawn. Therefore, larger, placebo-controlled clinical trials are warranted to determine if these initial observations can be substantiated, to investigate potential sex-specific and long-term effects, and to explore biomarkers that may help elucidate the mechanisms of the Neurotrophic peptide mixture in PD.

## Methods

### Patient information

The exploratory study included 17 patients with Parkinson’s disease with an established diagnosis according to the inclusion-exclusion criteria of the British Brain Bank^32^, aged 45–74 years with disease stage 2.0–3.0 (2.9 ± 0.3, according to Hoehn and Yahr scale). Written informed consent was obtained from all participants.

The neurotrophic peptide mixture was prescribed against the background of basic stable doses of antiparkinsonian therapy (Levodopa-containing drugs), which were used during 28–30 days before starting the course of neurotrophic peptide mixture and during the entire study period. No dose adjustments were allowed during the study, and medication dose was not included as a covariate in the statistical analysis. The median daily Levodopa dose was about 500 mg, it was individually optimized depending on disease stage and the patient’s condition. The Neurotrophic peptide mixture dosage regimen was 20 ml (215.2 mg/ml) intravenously, once a day for 10 days. Infusions were carried out in the first half of the day.

### Ethics statement and study design

The Ethics Committee of the Clinical Department of the Institute of Gerontology named after D.F. Chebotariov of the National Academy of Medical Sciences of Ukraine approved the protocol of current research (Protocol No. 4, 09/06/2022). Written informed consent was obtained from all participants prior to enrollment.

The study was conducted in accordance with the principles of the Declaration of Helsinki (2000) and the European Council Convention on Human Rights and Biomedicine (2005), and relevant national standards for the participation in the research were taken into account.

### Clinical assessment

Characterization of the stages of PD was carried out using the Hoehn and Yahr scale. To assess the dynamics of motor disorders, the standardized international Unified Parkinson’s Disease Rating Scale (UPDRS) was used. For a comprehensive assessment of the state of non-motor functions, the following clinical scales were used:

- Mini-Mental State Examination (MMSE);
- Test battery for assessing frontal dysfunction (Frontal Assessment Battery, FAB);
- Montreal Cognitive Assessment (MoCA);
- Beck Depression Inventory (BDI);
- Spielberger-Hanin Anxiety Scale.

### Sensorimotor reaction and motor tempo

Motor tempo was determined using a computer program as the time between consecutive presses of the same finger on two keys spaced 20 cm apart on the keyboard. The patient was instructed to press the keys at the fastest possible tempo. The duration of each test was 20 s. Testing was conducted for each hand separately, and then the data were averaged.

The latent period of simple sensorimotor reaction was determined as the interval between the moment of presentation of a visual signal on the computer monitor and the moment the patient pressed the key in response to the signal. Geometric shapes (circle, square, oval) of red and white colors were used as signals, with 20 signals presented at intervals of 2–4 s. Testing was conducted for each hand separately, and the data were averaged.

### Electroneuromyography

The technique of registering the H-reflex, as a reflex response of muscles to the stimulation of sensory fibers of a mixed nerve followed by monosynaptic activation of spinal motoneurons and nerve motor fibers, was performed using disposable surface recording electrodes placed in the projection of the posterior group of muscles of the calf, with stimulation of the tibial nerve in the popliteal fossa. The dynamics of the amplitudes of H- and M-responses (mV) with increasing strength of nerve electrical stimulation (mA), the threshold for the occurrence of the H-reflex (mA), the latency period of the H-reflex (ms), and the ratio of the maximum amplitude of H max/M max (%) were evaluated.

### Evoked potentials P300

EEG was recorded monopolarly from 21 leads (Fp1, Fp2, Fpz, F3, F4, Fz, C3, C4, Cz, P3, P4, Pz, T3, T4, F7, F8, T5, T6, O1, O2, Oz). The combined ear electrode was used as a reference electrode. The interelectrode resistance was less than 5 kOhm, the signal sampling frequency was 500 Hz, and the high- and low-pass filters were 0.3 Hz and 35 Hz, respectively. Endogenous cognitive evoked potentials N2 and P3 were recorded according to the oddball paradigm using sound stimuli with a frequency of 1000 and 2000 Hz and a duration of 10 ms.

### Platelet and mitochondrial structure with Electron microscopy

Blood plasma enriched with platelets containing leukocytes was obtained by centrifugation of whole blood obtained from the ulnar vein of the subjects at room temperature for 15 min at 120 g in a T-30 centrifuge. The plasma was carefully separated from the sediment and centrifuged at 2000 g for 20 min using a Vortecs Combispin FVL-2400N minicentrifuge^33^.

Electron microscopic studies of platelets and mitochondria were carried out according to the generally accepted method with double fixation with glutaraldehyde and OSO_4_, dehydration with alcohols of increasing concentrations and subsequent embedding in Epon. Ultrathin sections with a thickness of 40–60 nm, contrasted with uranyl acetate and lead citrate, were examined using an electron microscope PEM-125K (Ukraine).

Morphometric calculations of platelets’ structure were performed using the Image Tool (USA) on 130–150 fields for each group of subjects. The number of optically dense small σ-granules and optically transparent large α-granules was determined.

### PBMC isolation and quantitative PCR

Blood collection, PBMC isolation, RNA extraction and cDNA synthesis were performed by standard methods. The collection of blood for further cell isolation and qPCR was made on 0 and 11th day (after a 10-day course of neurotrophic peptide mixture treatment). Briefly, patients’ blood was collected in 4 ml vacutainers with EDTA. Density gradient centrifugation of diluted whole blood layered over a density gradient medium was used for PBMC isolation. PBMC were gradually frozen at a temperature of −20 °C, and then transferred to the temperature of −80 °C for long-term storage. RNA was isolated from cells using the Direct-zol RNA Miniprep Kit (Zymo Research, USA) following the manufacturer’s protocols. The isolated RNA was treated with DNase to remove DNA residues that could give a false positive signal for the expression of target genes. After this, reverse transcription was carried out using a commercial kit ZymoScript RT PreMix (Zymo Research, USA), guided by the manufacturer’s recommendations.

Samples of this cDNA were used for further qPCR-RT (quantitative real-time polymerase chain reaction), which was used to measure the expression of the *DJ-1*, *PINK1, BDNF* and *TNF*α genes. To identify a suitable internal control for RT-qPCR data normalization, we empirically tested the expression stability of three candidate reference genes (*rps18*, *gusb*, and *beta-actin*). The sequences of all used primers are listed in the Supplementary Information. Analysis of the raw Cqt values revealed that both *beta-actin* and *gusb* were the most stably expressed genes, showing minimal variation and a strong positive correlation with each other. In contrast, *rps18* expression was found to be significantly more variable across our samples and was therefore excluded. Although both *beta-actin* and *gusb* were suitable candidates, we selected *beta-actin* for normalization due to its slightly higher expression stability.

The sequences of all used primers are listed in the Supplementary Information. The PCR reaction mix was prepared using a commercial kit of reagents for RT-PCR (HOT FIREPol® EvaGreen® qPCR Mix Plus, Solis BioDyne, Estonia) - reverse transcription and polymerase chain reaction with the addition of betaine (Sigma, USA) at a final concentration of 1 M. To determine differences in mRNA expression of Parkin genes, Repeated Measures Anova was used.

Accumulation curves were generated using Opticon Monitor 3 software. After amplification was completed, raw gene product amplification data were used to calculate Cy0.

### Measurement of oxidative stress biomarkers

Secondary products of lipid peroxidation, the activity of antioxidant enzymes superoxide dismutase (SOD) and catalase (CAT) were measured in blood plasma. In red blood cells – the content of hydrogen peroxide (H2O2); the state of the glutathione system was assessed by the content of reduced glutathione (GSH) and the activity of the enzyme glutathione peroxidase (GP).

For erythrocyte isolation, samples were centrifuged at 8000 rpm for 15 min to separate plasma and red blood cells. Using centrifugation, erythrocytes were washed twice of plasma with cold sterile physiological sodium chloride solution. Erythrocyte hemolysate was obtained by adding distilled water to washed erythrocytes in a 1:1 ratio. The hemoglobin (Hb) content was also measured (Hemoglobin Assay kit MAK115, Sigma-Aldrich, St. Louis, MO, USA).

Determination of thiobarbituric acid reactive substances (TBARS) levels: TBARS were isolated by boiling plasma with the thiobarbituric acid reagent (0.5% 2-thiobarbituric acid/10% trichloroacetic acid/0.63 mM hydrochloric acid) and measuring the absorbance at 532 nm. The results are expressed as µM/ml using a molar extinction coefficient 1.56 * 10^5 ^mm^−1^ cm^−1^.

Catalase activity measurement: The method is based on the ability of molybdenum salts to form a colored complex with hydrogen peroxide, which was determined at a wavelength of 410 nm. Enzyme activity (μM/min/L) was calculated using a molar extinction coefficient of 22.2 * 10^3 ^mm^−1^cm^−1^.

Superoxide dismutase (SOD) activity was determined spectrophotometrically. The method is based on the ability of SOD to inhibit the autoxidation of adrenaline at pH 10.2. The results were expressed as specific activity of the enzyme in units per ml of plasma. One unit of SOD activity is defined as the amount of protein causing 50% inhibition of the conversion rate of adrenaline to adrenochrome under specified conditions.

The activity of selenium-dependent glutathione peroxidase (GPx) was determined by recording the rate of NADPH oxidation in the presence of reduced glutathione at a wavelength of 340 nm. Enzyme activity was expressed in nM NADPH/min/ml.

The amount of reduced glutathione (μM/ml) was determined using Elman’s reagent (5.5’-dithio-bis-nitrobenzoic acid in absolute ethanol). The change in extinction was recorded at a wavelength of 412 nm every minute for 5 minutes.

Hydrogen peroxide content was studied using the FOX method, which is based on the peroxide-mediated oxidation of Fe2 + , followed by the reaction of Fe3+ with xylenol orange. Absorbance of the Fe3 + -xylenol orange complex was determined at a wavelength of 560 nm. The amount of H_2_O_2_ (μM/mgHb) was calculated using the H_2_O_2_ standard curve for each independent experiment.

### Machine learning prediction of UPDRS score change

To model UPDRS score changes, H2O’s AutoML framework was employed^34^, allowing for the training, tuning, and evaluation of multiple Machine Learning models, including Gradient Boosting Machines (GBMs) and Random Forests (RFs). AutoML facilitated hyperparameter optimization, ensuring robust and accurate predictions.

- Gradient Boosting Machines (GBMs): GBMs iteratively build a series of decision trees, where each subsequent tree focuses on correcting errors made by its predecessors. This sequential approach enables GBMs to capture intricate, non-linear relationships within the data, making them particularly suitable for analyzing how clinical and biochemical factors impact UPDRS score changes and, consequently, therapeutic response^35^.
- Random Forests (RFs): RFs are ensemble models that combine multiple decision trees, each trained on a random subset of the data. Known for their robustness and reduced susceptibility to overfitting, RFs provide valuable insights into feature importance by averaging across numerous trees^36^. This model serves as a stable baseline to complement the more flexible GBMs.

The models were evaluated using the following metrics to quantify prediction accuracy:

- Root Mean Squared Error (RMSE): Captures the average deviation between predicted and actual values.
- Mean Squared Error (MSE): Measures the variance of predictions relative to actual outcomes.
- Mean Absolute Error (MAE): Reflects the average absolute deviation between predicted and observed values.

These metrics allowed for a thorough assessment of each model’s ability to predict UPDRS score changes, highlighting the Neurotrophic peptide mixture’s potential impact on disease progression.

Feature importance was evaluated using the top 10-performing models from a set of 50 trained with H2O AutoML. Each feature’s importance was measured by its “scaled importance,” which represents its relative contribution to prediction accuracy. The models were trained using two-fold cross-validation, and feature importance was extracted during the validation stage.

Data from the top 10 models were aggregated, and features were sorted by their median feature importance. It is worth noting that while building a generalized model on such a small dataset is not feasible, the features identified with the highest median importance could be considered potential markers in future studies. The results were visualized using a boxplot (Fig. 16) to highlight the median importance and variability of each feature.

### Statistical analysis

Hypotheses about the effects of the drug were tested using the Wilcoxon (for related samples) and Mann-Whitney (for independent groups) tests. The relationship between the investigated indicators was determined in the form of Spearman’s rank correlation coefficients.

Statistical processing of gene expression data was performed using Statistica 8.0 software (StatSoft Inc., USA). For statistical analysis, Spearman’s non-parametric test was used to investigate the correlation between parameters. The difference in expression of the studied genes before and after treatment was determined using the Wilcoxon *t*-test (Wilcoxon signed-rank test).

The results of the study of the state of the prooxidant-antioxidant system were processed statistically using the Origin program. The significance of differences between comparison groups was determined by the method of analysis of variance (ANOVA) followed by the Bonferroni test (post-hoc test).

### Outcomes and hierarchy

For this exploratory study, outcomes were grouped into three hierarchical levels.

- Clinically central endpoints were prespecified as the UPDRS III (motor score) and the UPDRS Total score, as the primary efficacy measures, as these are the most widely accepted, clinically relevant, and objective indicators of motor impairment and overall disease severity in Parkinson’s disease.
- Secondary clinical endpoints included UPDRS I (non-motor experiences of daily living) and UPDRS II (motor experiences of daily living), and validated scales of non-motor function: MMSE, MoCA, FAB (cognitive measures), and BDI and Spielberger–Hanin Anxiety Scale (mood and affective measures). These outcomes were considered supportive of the clinically central endpoints.
- Exploratory outcomes included additional non-motor measures, neurophysiological parameters (P300 latency and amplitude), and molecular/structural biomarkers (gene expression, platelet ultrastructure, mitochondrial morphology, and oxidative stress markers). These outcomes were included to generate mechanistic insights into the potential modes of action of the Neurotrophic peptide mixture.

Formal hypothesis testing was restricted to the clinically central endpoints. Secondary and exploratory outcomes are presented with descriptive statistics and effect sizes. To minimize Type I error, no multiple-testing correction was applied outside of the clinically central endpoints.

Correlation analyses were performed using Spearman’s rank correlation coefficient (ρ). For correlations involving clinically central endpoints, *p*-values are reported. Correlations among secondary and exploratory outcomes are reported without formal inferential testing, to avoid Type I error inflation.

## Supplementary information

Supplementary Information

## Data Availability

The datasets used and/or analyzed during the current study are available from the corresponding author on reasonable request.
