# Supplementary material for: Evaluation of the neurotrophic peptide mixture in pathogenetic therapy of patients with Parkinson’s disease
Source: NPJ Parkinsons Dis. 2026 Jan 23;12:55. doi: 10.1038/s41531-026-01270-6 (PMC12932723; doi:10.1038/s41531-026-01270-6)
Supplement: Supplementary file 1 — Supplementary Information [file 41531_2026_1270_MOESM1_ESM.docx]

**Evaluation of neurotrophic peptide mixture effectiveness in pathogenetic therapy of patients with Parkinson’s disease**

Dmytro Krasnienkov^1,2^, Iryna Karaban^1^, Nina Karasevych^1^, Nataliia Melnyk^1^, Sergiy Kryzhanovskyi^1^, Kateryna Rozova^3^, Olga Gonchar^3^, Iryna Mankovska^3^, Sofiia Smovzh^1,4^, Kostiantyn Midlovets^1,5^, Olexiy Barsukov^1,6^, Oksana Zabuha^1^, Tetiana Papurina^1,4^

1. D.F. Chebotarev Institute of Gerontology of the National Academy of Medical Sciences of Ukraine, 67, Vyshgorodska Str., 04114 Kyiv, Ukraine.
2. Blackthorn AI, Ltd, London, United Kingdom.
3. Bogomoletz Institute of Physiology of the National Academy of Sciences of Ukraine, 4, Bohomoltsia Str., 01601 Kyiv, Ukraine.
4. Preci LLC, 37B, Kyivska Street, 09100 Bila Tserkva, Ukraine.
5. V. N. Karazin Kharkiv National University, [4, Svobody Sq,](https://www.google.com/maps/search/4+Svobody+Sq,+Kharkiv?entry=gmail&source=g) 61022 [Kharkiv](https://www.google.com/maps/search/4+Svobody+Sq,+Kharkiv?entry=gmail&source=g), Ukraine.
6. M.H. Kholodny Institute of Botany, 2, Tereshchenkivska Str., 01004 Kyiv, Ukraine.

Correspondence to: Tetiana Papurina

E-mail: tatyana_papurina@ukr.net

Supplementary Table 1.

Forward and Reverse Sequences for candidate reference and target genes.

| Genes | Forward Sequence (5´→3′) | Reverse Sequence (3´→5′) |
| --- | --- | --- |
| Candidate reference genes | | |
| GusB | GCC AAT GAA ACC AGG TAT CCC | GCT CAA GTA AAC AGG CTG TTT TCC |
| RPS18 | TAG CCT TTG CCA TCA CTG CC | CAT GAG CAT ATC TTC GGC CC |
| β-Actin | GAG CTA CGA GCT GCC TGA | GGA TGC CAC AGG ACT CCA |
| Target genes | | |
| TNF-α | CTC TTC TGC CTG CTG CAC TTT G | ATG GGC TAC AGG CTT GTC ACT C |
| BDNF | TGC AGG GGC ATA GAC AAA AGG | CTT ATG AAT CGC CAG CCA ATT CTC |
| DJ‐1 | CTG GCT AAA GGA GCA GAG GA | ATC TTC AAG GCT GGC ATC AG |
| PINK1 | CAA GAG AGG TCC CAA GCA AC | GGC AGC ACA TCA GGG TAG TC |

​
